# A DNA Nanomachine Modulates the Stemness-Associated Signaling Pathways for Overcoming Chemoresistance by Temporally Programming Drug Release

**DOI:** 10.34133/research.0999

**Published:** 2025-12-09

**Authors:** Jie Chen, Xiaodie Li, Qian Chen, Xuyang Zhou, Jialin Zeng, Linlang Guo, Yinan Zhang, Dayong Yang, Chao Zhang

**Affiliations:** ^1^ Department of Oncology, Zhujiang Hospital, Southern Medical University, Guangzhou 510282, P. R. China.; ^2^ Department of Pathology, Zhujiang Hospital, Southern Medical University, Guangzhou 510282, P. R. China.; ^3^ Department of Radiation Oncology, Cancer Hospital of Shantou University Medical College, Shantou 515041, P. R. China.; ^4^ Department of Medical Oncology, Sun Yat-Sen University Cancer Center, Guangzhou 510060, P. R. China.; ^5^ School of Chemical Science and Engineering, Tongji University, Shanghai 200092, P. R. China.; ^6^ Department of Chemistry, State Key Laboratory of Molecular Engineering of Polymers, Shanghai Key Laboratory of Molecular Catalysis and Innovative Materials, College of Chemistry and Materials, Fudan University, Shanghai 200438, P. R. China.

## Abstract

Chemoresistance is a primary cause of cancer treatment failure, due to the lack of specific regulatory strategies arising from unclear mechanisms. Here, we uncover the pivotal role of the PRMT1/SOX2 axis in regulating cancer stemness, a key factor contributing to cancer chemoresistance. In light of this, we construct a DNA nanomachine (DNM) to overcome chemoresistance by reversing cancer stemness. This DNM is constructed using a programmable DNA origami framework, incorporating CD44-targeting aptamers and glutathione (GSH)-responsive stemness inhibitors (DCLX069) as functional components. The DNM exhibits a specific affinity toward CD44-overexpressing tumor cells, enabling the effective delivery of the loaded cisplatin (CDDP) to the tumor cells. Upon entering the tumor cells, DCLX069 is rapidly released from the DNM due to high intracellular GSH levels, leading to swift regulation of the PRMT1/SOX2 axis. In contrast, CDDP exhibits a gradual enzymatic release profile. This temporally programmed release enables the reversal of cancer stemness before chemotherapy initiation, resulting in a substantial improvement in CDDP chemosensitivity and a significant increase in the median survival of tumor-bearing mice from 27 to over 56 d with DNM assistance. This study highlights the promising potential of this DNA nanotechnology-empowered therapy in addressing chemoresistance in malignant tumors.

## Introduction

Chemoresistance, the phenomenon where tumor cells display innate or acquired resistance to one or more chemotherapeutic drugs, is the primary cause of cancer treatment failure in clinical practice [1–3]. This resistance often results in increased local invasion, metastasis, and a poor prognosis, highlighting the crucial importance of elucidating the mechanisms behind chemoresistance [4]. The limited efficacy of delivering chemotherapeutic drugs to tumor cells is a key contributor to chemoresistance [5–8]. Nonetheless, chemoresistance is a complex phenomenon influenced by various biological characteristics regulated by intrinsic cell signaling networks [9,10]. Therefore, a thorough comprehension of the biological characteristics behind chemoresistance is essential for exploring potential treatment strategies to overcome it [11].

Cancer stemness denotes the unique biological characteristics of unlimited self-renewal and differentiation in a subset of cancer cells [12–14]. This characteristic enables cancer cells to resist the effect of chemotherapy, ultimately leading to incomplete tumor clearance and further recurrence [15–17]. Thus, cancer stemness is widely recognized as a critical driver of chemoresistance [18]. While increasing evidence suggests that the aberration of specific genes can bolster cell stemness by forming feedback loops and sustained activation of carcinogenic signaling pathways [19–22], the identified stemness-associated genes remain disproportionately low compared to the multitude of intracellular genes. Moreover, unraveling the regulatory mechanisms and devising efficacious strategies to counteract stemness present formidable challenges.

**Fig. 1.**
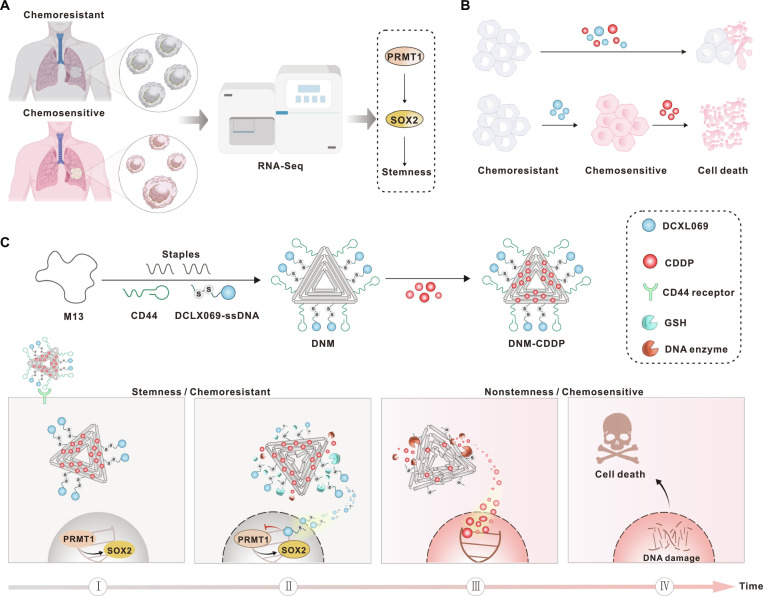
Schematic illustration of a DNM-empowered programmed release strategy to overcome stemness for reversing chemoresistance and reinforcing anticancer efficacy. (A) Schematic representation of the discovery process of the stemness-associated PRMT1-SOX2 signaling pathway. (B) Schematic representation of the greater treatment efficacy of the sequential administration than the simultaneous administration of DCLX069 and CDDP. (C) Schematic depicting the fabrication process of DNM-CDDP and the mechanism of action of DNM-CDDP: (I) DNM-CDDP specifically targets cancer cells and (II) releases DCLX069 responsively to the intracellular high-level GSH at the first step to block the PRMT1-SOX2 signal pathway, contributing to reduced stemness and enhanced chemosensitivity; (III) DNA enzymes digest DNM and CDDP is released in chemosensitive cells with reduced stemness, (IV) efficiently triggering DNA damage and apoptosis against the tumor cells.

In this study, we demonstrated the important role of protein arginine methyltransferase 1 (PRMT1) in promoting chemoresistance in small cell lung cancer (SCLC) by reinforcing cancer stemness through the PRMT1-SOX2 signaling pathway, suggesting PRMT1 as a promising target for reversing stemness (Fig. 1A). Additionally, our cell counting kit-8 (CCK-8) assay revealed that a sequential administration of DCLX069 [23,24] (a PRMT1 inhibitor) followed by cisplatin (CDDP) exhibited greater treatment efficacy than the simultaneous administration of DCLX069 and CDDP (Fig. 1B). This finding underscores the significance of reducing stemness before chemotherapy initiation. Based on these findings, a DNA nanomachine (DNM) was designed to counteract cancer stemness, combat chemoresistance, and enhance the therapeutic efficacy of CDDP. The synthesis process and underlying mechanism are detailed in Fig. 1C. Specifically, a programmable triangular DNA origami structure was assembled to serve as the building block of the DNM, with CD44-targeting aptamers and glutathione (GSH)-responsive DCLX069 as the functional components of the DNM. The DNM exhibited targeted binding to CD44-overexpressing SCLC cells, facilitating the precise delivery of the loaded CDDP to the tumor cells. Upon entering the tumor cells, DCLX069 was rapidly released from the DNM in response to elevated intracellular GSH levels, leading to a robust modulation of the PRMT1/SOX2 axis. In contrast, CDDP was released gradually through enzymatic hydrolysis. This programmed release strategy allowed time to reverse cancer stemness before chemotherapy initiation. Consequently, the median survival of tumor-bearing mice was extended from 27 to over 56 d, demonstrating the substantial improvement in CDDP chemosensitivity after DNM treatment. It is anticipated that this innovative DNM-based therapeutic approach has the potential to revolutionize the current treatment landscape for not only the SCLC but also other malignant tumors.

## Results and Discussion

### PRMT1-SOX2 signaling pathway drives chemoresistance in SCLC

**Fig. 2.**
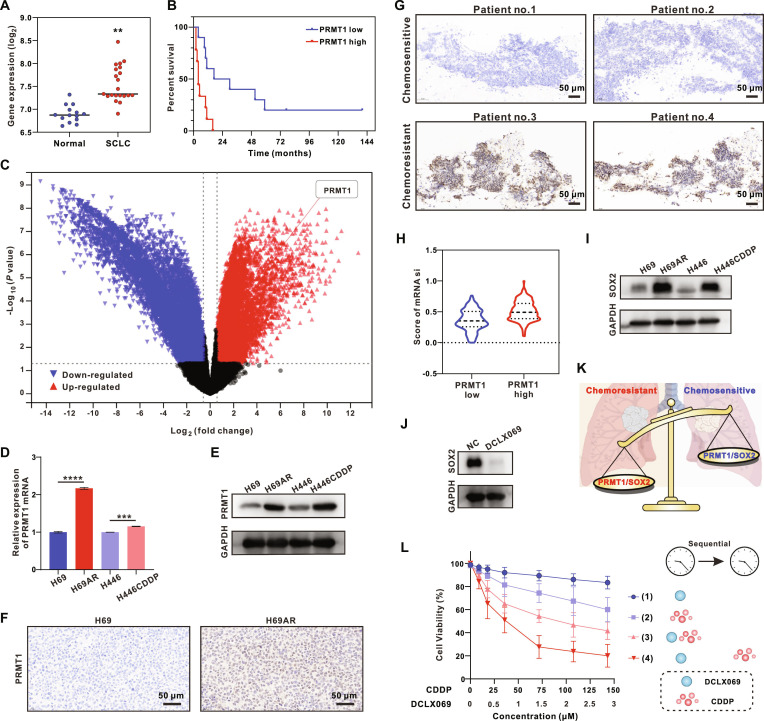
PRMT1 expression is up-regulated in SCLC patients and induces poor outcomes and resistance to chemotherapy by activating the SOX2 signaling pathway. (A) Expression of PRMT1 between SCLC patients and healthy people. (B) Kaplan–Meier survival curve of PRMT1 high-expressed population and PRMT1 low-expressed population in SCLC patients (*P* = 0.0023). (C) RNA sequencing analysis of drug-resistant (H69AR) sublines relative to drug-sensitive (H69) sublines. (D and E) RT-qPCR (D) and Western blot analysis (E) of PRMT1 expression in drug-sensitive (H69 and H446) and drug-resistant (H69AR and H446CDDP) sublines. (F) IHC images of the PRMT1 expression in tumor tissues of H69AR and H69 tumor-bearing nude mice. (G) IHC images of the PRMT1 expression in tumor tissues of clinical SCLC patient specimens. (H) Score of mRNAsi in lung cancer patients with high or low PRMT1 expression. (I) Western blot analysis of SOX2 expression in drug-sensitive (H69 and H446) and drug-resistant (H69AR and H446CDDP) sublines. (J) Western blot analysis of SOX2 expression in H69AR cells after 12 h of DCLX069 treatment. (K) Schematic representation of the PRMT1-SOX2 signaling pathway in chemosensitive and chemoresistant SCLC patients. (L) Viabilities of H69AR cells via CCK-8 assay after different treatments: (1) DCLX069 treatment for 24 h; (2) CDDP treatment for 24 h; (3) cotreatment with DCLX069 and CDDP for 24 h; (4) pretreatment with DCLX069 for 24 h followed by CDDP cotreatment for an additional 24 h. Data are shown as mean ± SD (*n* = 3, **P < 0.01, ***P < 0.001, ****P < 0.0001).

We conducted a comparative analysis of genetic expression profiles between SCLC patients and healthy controls to uncover the critical genes involved in SCLC pathogenesis. Our findings revealed a significant up-regulation of PRMT1 in SCLC patients (Fig. 2A), which was consistent with previous studies [25,26]. Single-cell transcriptomic analysis further confirmed that PRMT1 exhibits high expression in tumor cells of SCLC (Figs. S1 and S2A and B). Subsequent analysis indicated a correlation between elevated PRMT1 expression and diminished survival prospects in SCLC patients (Fig. 2B). This finding suggests that PRMT1 may serve as both a prognostic indicator and a potential therapeutic target for SCLC. To elucidate the underlying mechanism, we explored the association between PRMT1 expression and chemoresistance, the principal factor contributing to poor prognosis in SCLC [27]. RNA sequencing analysis of chemoresistant (H69AR) and chemosensitive (H69) SCLC sublines revealed markedly elevated expression of PRMT1 in H69AR cells (Fig. 2C). This finding was substantiated by reverse transcription quantitative polymerase chain reaction (RT-qPCR) (Fig. 2D) and Western blot analysis (Fig. 2E and Fig. S3A), which demonstrated increased PRMT1 expression in drug-resistant sublines (H446CDDP and H69AR) relative to drug-sensitive sublines (H446 and H69). Immunohistochemical (IHC) staining of subcutaneous tumor tissues from H69 and H69AR tumor-bearing mice further confirmed these findings (Fig. 2F). Similarly, clinical SCLC patient specimens from chemosensitive (newly diagnosed and untreated) and chemoresistant (recurrent) tumors displayed higher PRMT1 expression in chemoresistant tumors (Fig. 2G). Our findings strongly suggest that PRMT1 overexpression is associated with chemoresistance in SCLC and could serve as a predictive marker for SCLC prognosis.

We examined public databases to understand how PRMT1 contributes to drug resistance. Our analysis revealed that lung cancer patients with high PRMT1 expression also exhibited elevated mRNA expression-based stemness index (mRNAsi) scores [28–30], indicating increased cancer stemness (Fig. 2H). Single-cell transcriptomic analysis (Fig. S2C) confirmed that SOX2, a marker associated with stemness [31–33], exhibits high expression in tumor cells of SCLC. Additionally, our Western blot analysis indicated heightened SOX2 expression in drug-resistant SCLC sublines as opposed to drug-sensitive sublines (Fig. 2I and Fig. S3B). Furthermore, treatment with DCLX069 (Fig. S4A), a small-molecule inhibitor of PRMT1, considerably reduced SOX2 expression in H69AR cells ((Fig. 2J). These findings suggest that PRMT1 inhibition reduces cancer stemness in SCLC, potentially through the PRMT1-SOX2 signaling pathway (Fig. 2K).

Based on these results, we hypothesized that the PRMT1 inhibitor DCLX069 could help reverse chemoresistance in SCLC by reducing cancer stemness. To test this hypothesis, we investigated the cytotoxic effects of combining CDDP with DCLX069 on drug-resistant SCLC sublines. CCK-8 assays demonstrated that DCLX069 alone exhibited a modest antitumor effect against H69AR cells. Even when incubated at a concentration of 3 μM for 48 h, the cell viability remained at 86% (Fig. S4B). The combination of DCLX069 with CDDP significantly increased cytotoxic effects against H69AR cells compared to CDDP treatment alone, with the percentage of cell viability reduced from 60% to 42% (Fig. 2L). Moreover, pretreatment with DCLX069 followed by cotreatment with CDDP resulted in further increased cytotoxicity, reducing the percentage of cell viability to 20%. This indicates a more pronounced anti-chemoresistant effect. These findings suggest that pretreatment with DCLX069 effectively reduces cancer stemness and substantially enhances the susceptibility of SCLC cells to chemotherapeutic drugs.

### Preparation and characterization of DNM and DNM-CDDP

**Fig. 3.**
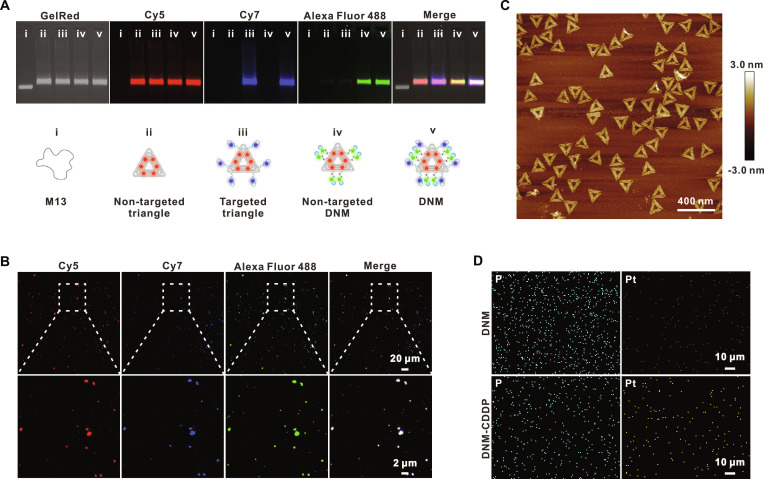
Preparation and characterization of DNM and DNM-CDDP. (A) Fluorescence agarose gel electrophoresis analysis of DNM and control groups. (B) Fluorescence colocalization images of the DNM. (C) AFM image of the DNM. (D) EDS element maps for P and Pt of DNM and DNM-CDDP.

Initially, a triangular DNA origami (Fig. S5A) was constructed using a previously described method [34–38] as the foundational component for DNM. Subsequently, a CD44-targeting aptamer and a GSH-responsive DCLX069 were integrated into the triangular DNA origami, functioning as the key elements of DNM. To confirm the successful assembly of these functional components into DNM, fluorescence agarose gel electrophoresis analysis (Fig. 3A) and fluorescence colocalization assay (Fig. 3B) were conducted. The colocalization of the fluorescence signals from the Cy5-labeled triangle DNA origami (red), Cy7-labeled CD44 aptamers (blue), and Alexa Fluor 488-labeled DCLX069-ssDNA (single-stranded DNA) (green) validated the successful assembly of DNM. Atomic force microscopy (AFM) imaging revealed the triangular configuration of the DNM, in which its functional components remained indistinguishable owing to limited absorption on the mica substrate (Fig. 3C and Fig. S5B). CDDP was then loaded into DNM to construct DNM-CDDP. Energy-dispersive spectroscopy (EDS) was utilized to evaluate the loading of CDDP into DNM, revealing a notably higher amount of elemental platinum (Pt) in DNM-CDDP compared to DNM. However, there were no discernible disparities in the levels of elemental phosphorus (P) between the 2, signifying the effective loading of CDDP (Fig. 3D). Notably, the structure of DNM remained unaltered post-CDDP loading, as validated by AFM analysis (Fig. S5C). The DNA sequences of the staple strands used for assembling DNM and DNM-CDDP, along with those of control groups, can be found in Tables S1 to S4.

### Investigation of the temporally programmed release of cargos

**Fig. 4.**
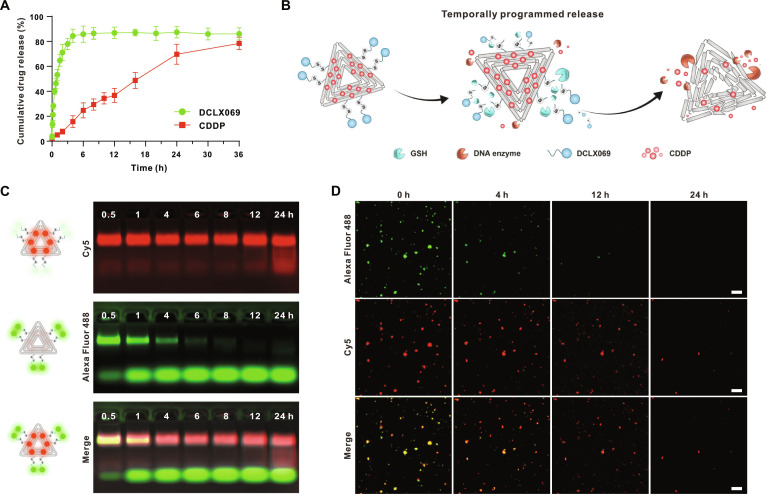
Investigation of the temporally programmed release of cargos. (A) In vitro cumulative drug release efficiency of DNM-CDDP. (B) Schematic illustration of the temporally programmed release of DCLX069 and CDDP. (C) Fluorescence agarose gel electrophoresis analysis of the stability and temporally programmed cargo release of Cy5/Alexa Fluor 488-colabeled DNM incubated in tumor tissue lysate for 0.5, 1, 4, 6, 8, 12, and 24 h. (D) Fluorescence colocalization images of Cy5/Alexa Fluor 488-colabeled DNM incubated in tumor tissue lysate for 0, 4, 12, and 24 h. Scale bars, 2 μm (*n* = 3, **P < 0.01, ***P < 0.001, ****P < 0.0001).

Following the successful synthesis of DNM-CDDP, our subsequent objective was to investigate the drug release kinetics of DNM-CDDP. After subjecting DNM-CDDP to tumor tissue lysate for varying durations, the release rates of DCLX069 and CDDP were detected through ultraviolet–visible (UV–Vis) spectroscopy and inductively coupled plasma mass spectrometry (ICP-MS). The results revealed a significant delay in the release of CDDP compared to DCLX069 (Fig. 4A). Specifically, DCLX069 exhibited a rapid release profile, with over 80% of the drug released within the first 4 h of incubation, followed by reaching a plateau phase. In contrast, CDDP exhibited a relatively slower release, releasing only ~15% within 4 h. Based on these results, we hypothesized that DCLX069 was released rapidly from the DNM in response to the high levels of GSH in tumor cells [39], while CDDP was released slowly from the DNM due to gradual enzymatic degradation of the DNA framework by cellular deoxyribonucleases (Fig. 4B).

To validate this hypothesis, we covalently linked Cy5 to the DNA origami. Given that CDDP was also covalently attached to the DNA origami [40,41], the release of Cy5 serves as a model for the release of CDDP. Additionally, we replaced DCLX069 with Alexa Fluor 488 and conjugated it to the ssDNA to monitor the release of DCLX069. The release processes of Cy5 and Alexa Fluor 488 were analyzed through fluorescence agarose gel electrophoresis (Fig. 4C) and fluorescence colocalization assays (Fig. 4D). The results showed that after incubating with tumor tissue lysate for 1 h, a distinct DNA band with strong green fluorescence emerged. This indicates the release of Alexa Fluor 488, suggesting that DCLX069 was rapidly detached from the DNM. Additionally, the Cy5 fluorescence signal was primarily concentrated in the initial DNA band, confirming the sturdy structure of the DNA origami (Fig. 4C). Therefore, we propose that CDDP could be retained on the DNA origami in the short run. The fluorescence colocalization assay corroborated this finding (Fig. 4D) by demonstrating a decrease in the colocalized relationship of red and green fluorescence signals over time. These findings indicate that DCLX069 was rapidly released from DNM in response to GSH, whereas CDDP was released more slowly following the degradation of the triangular DNA origami. This temporally programmed release behavior enables sufficient time for reversing cancer stemness prior to the initiation of chemotherapy.

### Evaluation of tumor-targeting ability of DNM

**Fig. 5.**
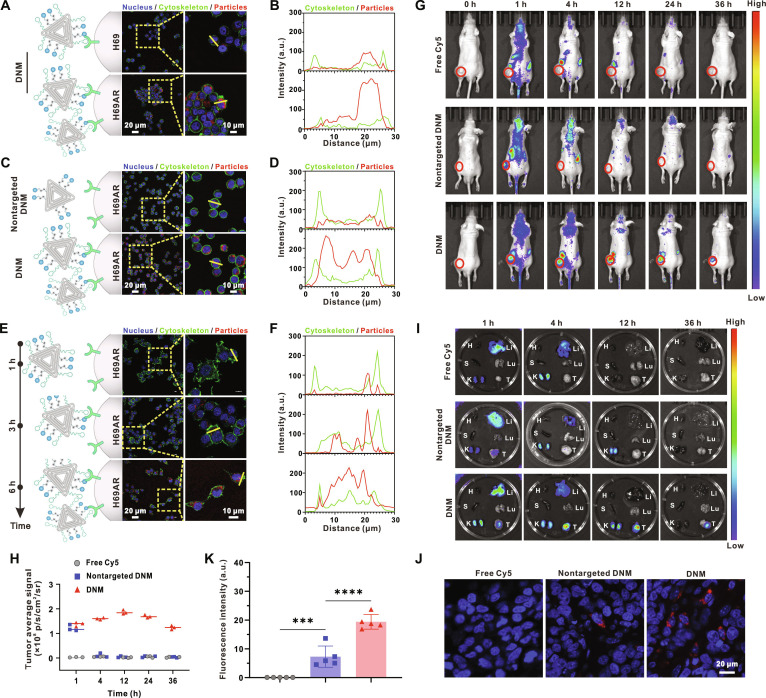
Investigation of the tumor-targeting capability of DNM in vitro and in vivo. (A) Confocal images of H69 and H69AR cells after incubation with DNM (Cy5-labeled DNM, red; FITC-labeled phalloidin, green; DAPI-labeled cell nucleus, blue; similarly, hereinafter). (B) Colocalization analysis of fluorescence profiles along the yellow lines in (A). (C) Confocal images of H69AR cells after incubation with DNM and nontargeted DNM (both labeled with Cy5). (D) Colocalization analysis of fluorescence profiles along the yellow lines in (C). (E) Confocal images of H69AR cells after incubation with DNM (labeled with Cy5) for various times. (F) Colocalization analysis of fluorescence profiles along the yellow lines in (E). (G) In vivo fluorescence distribution in H69AR tumor-bearing nude mice at the indicated time points after tail vein intravenous administration with free Cy5, DNM, and nontargeted DNM (both labeled with Cy5), respectively (0 h = before injection, tumors are indicated by red circles). (H) Quantitative analysis of the tumor fluorescence intensity in free Cy5, Cy5-labeled DNM, and nontargeted DNM groups at 12 h after injection. (I) Ex vivo representative fluorescence images of major organs and tumors of the mice at the indicated time points. The mice were respectively administrated with free Cy5, DNM, and nontargeted DNM (both labeled with Cy5). H, heart; Li, liver; S, spleen; Lu, lung; K, kidney; T, tumor. (J) Immunofluorescence images of the tumor slice of the indicated groups. (K) Fluorescence intensity of Cy5 in (J). Data are shown as mean ± SD (*n* = 3, ***P < 0.001, ****P < 0.0001).

Given the prevalent expression of CD44 on tumor cells [42–45] and the specific binding affinity of the CD44 aptamer [46,47] toward CD44, our subsequent aim was to evaluate the tumor-targeting efficacy of the DNM both in vitro and in vivo. Flow cytometry analysis demonstrated a markedly higher CD44 expression in the drug-resistant H69AR subline compared to the drug-sensitive H69 counterpart (Fig. S6), providing a rationale for targeting chemoresistant SCLC with the DNM. Confocal imaging and fluorescence colocalization analysis confirmed a notably higher uptake of DNM in H69AR cells relative to H69 cells (Fig. 5A and B), substantiating its superior targeting capability for drug-resistant tumor cells. This observation was further supported by flow cytometry analysis (Fig. S7). To verify the role of the CD44 aptamer in the targeting capability of DNM, we further compared the cellular uptake of DNM and nontargeted DNM in H69AR cells. The results demonstrated that DNM achieved a substantially higher uptake, ultimately confirming the necessity of the targeting potential of the CD44 aptamer (Fig. 5C and D). A subsequent flow cytometry analysis corroborated these results (Fig. S8). Confocal imaging depicted a heightened uptake efficiency of DNM with prolonged incubation time (Fig. 5E and F). Additional corroboration was provided by a subsequent flow cytometry analysis (Fig. S9).

The in vivo tumor-targeting ability was appraised using an H69AR tumor-bearing nude mouse model. The animal imaging showcased fluorescence distribution at specific intervals following intravenous administration. As depicted in Fig. 5G, both the DNM and nontargeted DNM initially exhibited fluorescence signals at the tumor location. However, the signal from the nontargeted DNM dissipated rapidly, likely due to its lack of tumor-targeting affinity. In contrast, the targeted DNM demonstrated progressive accumulation within the tumor, reaching its peak concentration at 12 h post-injection. Quantitative analysis revealed that, at this time point, the targeted DNM accumulated in the tumor approximately 40-fold more efficiently than the nontargeted DNM (Fig. 5H). Notably, the fluorescence signal remained observable in the tumor sites of the DNM group at 36 h post-injection (Fig. 5G). Ex vivo fluorescence imaging (Fig. 5I) and immunofluorescence staining (Fig. 5J and K) of tumors concurred with these findings, further confirming the enhanced tumor targeting and accumulation of DNM. In summary, these outcomes underscore the superior tumor-targeting efficacy of DNM both in vitro and in vivo.

### In vitro anticancer effect of DNM-CDDP

**Fig. 6.**
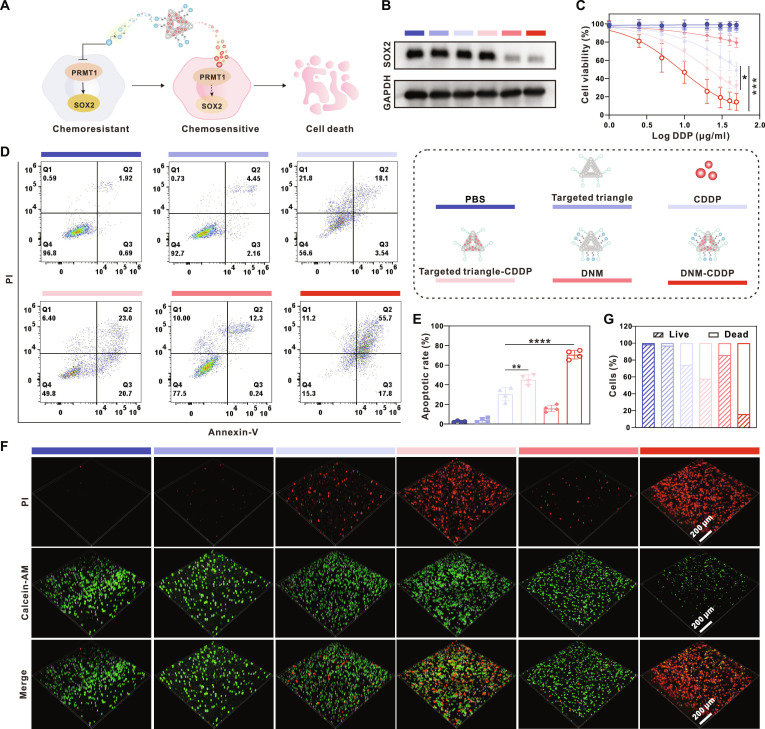
In vitro anticancer effect of DNM-CDDP against chemoresistant SCLC. (A) Schematic illustration of anticancer effect of DNM-CDDP against chemoresistant SCLC cells. (B) Western blot analysis of the SOX2 expression in H69AR cells after different treatments (PBS, targeted triangle, CDDP, targeted triangle-CDDP, DNM, DNM-CDDP) for 12 h. (C) Viabilities of H69AR cells after indicated treatments for 24 h. (D) Cell apoptosis analysis by flow cytometry of H69AR cells after indicated treatments for 24 h. (E) Apoptotic rate from (D). (F) Live-dead cell staining of H69AR cells after indicated treatments for 24 h. (G) Quantitative statistics of green (live) and red (dead) fluorescent signals by ImageJ analysis. Data are shown as mean ± SD (*n* = 3, *P < 0.05, **P < 0.01, ***P < 0.001, and ****P < 0.0001).

Having confirmed the tumor-targeting capability of DNM, our subsequent objective was to investigate whether DNM-CDDP could reduce the stemness, reverse chemoresistance, and render optimal anticancer efficacy against SCLC (Fig. 6A). Firstly, we assessed the stemness-reducing effect of DNM-CDDP by monitoring SOX2 expression in H69AR cells. As indicated in Fig. 6B and Fig. S10, the groups of phosphate-buffered saline (PBS), targeted triangle (triangular DNA origami equipped with CD44 aptamers), CDDP, and targeted triangle-CDDP (targeted triangle loaded with CDDP) showed no effect in reducing stemness. However, a significant decrease in SOX2 expression was observed after DNM and DNM-CDDP treatment, indicating stemness reversal facilitated by the timely release of DCLX069.

Next, we evaluated the chemoresistance-reversing effect of DNM-CDDP on SCLC in vitro*.* The viability of H69AR cells after exposure to different treatments was assessed using a CCK-8 assay. As shown in Fig. 6C, no obvious cytotoxicity was observed when cells were treated with targeted triangle and DNM. Relatively, CDDP exhibited some antitumor activity against H69AR cells, with a half-maximal inhibitory concentration (LogIC50) value of 1.741 μg/ml (Fig. S11). In comparison, the targeted triangle-CDDP showed an improved antitumor effect, resulting in a LogIC50 reduction to 1.331 μg/ml, possibly attributed to enhanced intracellular drug accumulation facilitated by the potent targeting ability of CD44 aptamers. Notably, the DNM-CDDP treatment group displayed the highest cytotoxicity, further reducing the LogIC50 to 0.9433 μg/ml, indicating its robust chemoresistance-reversing effect.

To further investigate the antitumor effect of DNM-CDDP, we conducted an apoptosis assay using flow cytometry and a live-dead cell staining assay with calcein-AM/propidium iodide (PI) double staining. The flow cytometry results revealed that DNM-CDDP treatment induced the highest level of apoptosis in H69AR cells (Fig. 6D and E). Calcein-AM/PI staining showed an increased prevalence of PI-stained cells in the DNM-CDDP group, indicating enhanced cell death (Fig. 6F and G). These findings demonstrate that DNM-CDDP effectively reduces stemness and reverses chemoresistance in SCLC cells, promoting their sensitivity to CDDP chemotherapy. Our results emphasize the potential of DNM-CDDP as a viable therapeutic option for overcoming chemoresistant SCLC cancer.

### In vivo anticancer effect of DNM-CDDP

**Fig. 7.**
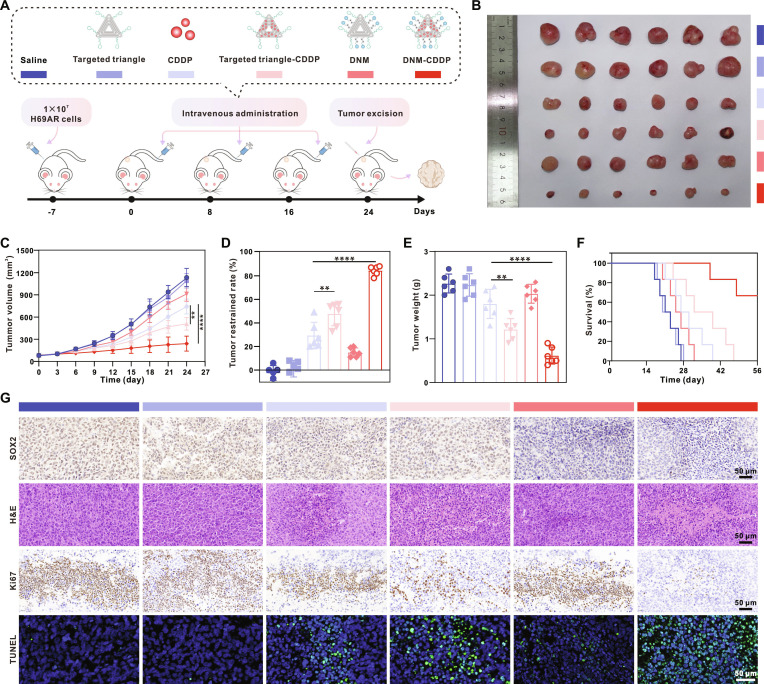
In vivo anticancer effect of DNM-CDDP against chemoresistant SCLC. (A) Schematic diagram of the in vivo therapeutic process of indicated treatments. (B) Images of the tumors collected from the mice at the 24th day post-initiation of the indicated treatments. (C) Tumor volume curves of the H69AR tumor-bearing nude mice after the indicated treatments. (D) Tumor restrained rate of different treatments. (E) Tumor weight data of the H69AR tumor-bearing mice 24 d after the indicated treatments. (F) Survival curves of H69AR tumor-bearing mice after the indicated treatments. (G) Representative images of the tumor sections examined by H&E, IHC, and immunofluorescent staining 24 d post-initiation of the indicated treatments. Data are shown as mean ± SD (*n* = 6, **P < 0.01 and ****P < 0.0001).

The in vivo anticancer effect of DNM-CDDP was initially assessed in SCLC tumor mouse xenograft models created through the inoculation of H69AR cells. Seven days post-inoculation, the mice were randomly divided into 6 groups and administered saline, targeted triangle, CDDP, targeted triangle-CDDP, DNM, and DNM-CDDP, respectively, via intravenous injection once every 8 d (Fig. 7A). Encouragingly, treatment with DNM-CDDP resulted in the highest inhibition of tumor growth, showing a significantly higher inhibitory effect compared to other treatments (Fig. 7B to E and Fig. S12). Survival curves revealed a significantly longer survival time in mice treated with DNM-CDDP compared to other groups (Fig. 7F). Notably, the median survival of the DNM-CDDP group was extended from 27 to over 56 d compared to the saline group. These findings demonstrate the efficacy of DNM-CDDP in inhibiting tumor growth and prolonging survival in mice bearing chemoresistant SCLC tumors.

To further investigate the combined therapeutic efficacy of DNM-CDDP, tumor tissues from all groups were collected and subjected to pathological analysis (Fig. 7G and Figs. S13 and S14). IHC assays revealed a significant reduction in the expression of the stemness-related protein SOX2 with DNM and DNM-CDDP treatment, indicating their abilities to reverse tumor stemness and enhance CDDP chemosensitivity. Hematoxylin and eosin (H&E) staining showed increased apoptosis and necrosis in DNM-CDDP-treated tumors. Ki67 IHC assays demonstrated significantly inhibited tumor cell proliferation after DNM-CDDP administration. Additionally, up-regulation of γ-H2AX, caspase-3, and TUNEL (terminal deoxynucleotidyl transferase-mediated deoxyuridine triphosphate nick end labeling) expression in the DNM-CDDP-treated group indicated significant DNA damage and apoptosis. These results align with the observed therapeutic outcomes in the in vivo antitumor experiment. Overall, these findings highlight the importance of the programmable drug release achieved by DNM-CDDP in enhancing the efficacy of remarkable chemosensitization and antitumor efficacy of DNM-CDDP in vivo against chemoresistant SCLC.

To assess the feasibility of clinical application of DNM-CDDP, we conducted a comprehensive biosafety evaluation encompassing body weight changes, detailed blood analyses, and histological examinations. Mice were randomized into 6 groups and administered saline, targeted triangle, CDDP, targeted triangle-CDDP, DNM, or DNM-CDDP (Fig. S15A) via intravenous injection. As shown in Fig. S15B, the CDDP treatment group exhibited notable weight loss compared to those receiving alternative treatments, signifying a heightened systemic toxicity level.

Routine hematological examinations were usually used to assess general and hematologic toxicities, while blood biochemistry tests were primarily used to evaluate cardiac, hepatic, and renal functions. In Fig. S15C, the CDDP group exhibited decreased white blood cell (WBC), neutrophil-to-lymphocyte ratio (Neut%), red blood cell (RBC), hemoglobin (HGB), and platelets (PLT), indicating significant hematologic toxicity. In Fig. S15D, elevated creatinine (CR) and blood urea nitrogen (BUN) levels in the CDDP group suggested apparent renal toxicity, which was consistent with previous studies [48,49]. Notably, treatment with DNM-CDDP did not result in hematologic or renal toxicities observed in the CDDP-alone group. This is likely attributed to the targeted delivery mechanism of DNM-CDDP, which effectively prevented excessive accumulation of CDDP in the bloodstream and kidneys. The early immunogenic response was assessed by quantifying the levels of interleukin-6 (IL-6), IFN-γ, and TNF-α in blood samples collected from the mice. As evident from the results in Fig. S15E, no immunogenic response was observed across the various treatment groups.

Additionally, conventional histological examination using H&E staining was performed on key organs (heart, liver, spleen, lung, and kidney) collected from nude mice to evaluate the organ-specific toxicity of CDDP and DNM-CDDP (Fig. S15F). The results showed some disorganization in the renal tissues of the CDDP-treated group, whereas no substantial pathological abnormalities were observed in the DNM-CDDP-treated group. The above results collectively demonstrate the favorable biosafety of DNM-CDDP, underscoring its potential as a promising therapeutic strategy for cancer treatment.

## Conclusion

In this study, we delved into the mechanisms underlying drug resistance in SCLC, identifying the pivotal role of the PRMT1/SOX2 axis in governing cancer stemness, a factor that substantially contributes to SCLC chemoresistance. Subsequently, we developed a DNM-based strategy aimed at modulating these mechanisms, ultimately affecting the reversal of drug resistance in SCLC. The DNM employs 2 distinct drug-release mechanisms within the tumor microenvironment. The PRMT1 inhibitor DCLX069 in DNM can be rapidly released in response to elevated intracellular GSH levels, leading to the disruption of the PRMT1-SOX2 pathway that reduces tumor cell stemness and enhances chemosensitivity. Conversely, the CDDP in DNM is gradually released with the enzymatic hydrolysis of DNM. The different release kinetics of the 2 agents create an optimal window for tumor cells to shift from a resistant to a sensitive state. This ultimately enhances the outcome of chemotherapy, leading to a significant increase in the median survival of tumor-bearing mice from 27 to over 56 d. Our study demonstrates a promising approach for the combined treatment of cancer and holds encouraging potential for clinical use.

The advantage of DNA materials lies in their high programmability, which endows them with abilities in precision diagnostics and treatment [50,51]. However, in the context of clinical translation involving DNA materials, the focus shifts to their biosafety and cost [52]. It is noteworthy that all the strands used to construct the DNM are inert and do not pose biosafety concerns. The results align with our expectations, showing that the DNM does not induce an immunogenic response in mice or have adverse effects on the function or pathological morphology of normal organs. Our study demonstrates a promising interdisciplinary approach to cancer treatment and holds encouraging potential for practical application.

## Materials and Methods

### Chemicals and reagent*s*

*N*-(3-(dimethylamino) propyl)-*N*′-ethylcarbodiimide hydrochloride (EDC) and *N*-hydroxysuccinimide (NHS) were purchased from China National Medicines Corporation Ltd. (Beijing, China). RPMI 1640 medium and fetal bovine serum (FBS) were purchased from Gibco (Waltham, WA, USA). Gel loading buffer and agarose were bought from Bio-Rad (Shanghai, China), while gel red was bought from US Everbright Inc. (Suzhou, China). M13mp18 ssDNA (7249) was purchased from Bioruler Ltd. (Shanghai, China), while labeled or unlabeled DNA staple strands were purchased from Sangon (Shanghai, China). CCK-8, Annexin V-FITC/PI Apoptosis Detection Kit, Live/Dead Viability Assay Kit, and Colorimetric TUNEL Apoptosis Assay Kit were all purchased from Beyotime (China). 4′,6-Diamidino-2-phenylindole, dihydrochloride (DAPI) and fluorescein isothiocyanate (FITC) phalloidin were acquired from Solarbio (Beijing, China). PRMT1 monoclonal antibody was purchased from Proteintech (Wuhan, China). SOX2 monoclonal antibody was purchased from Zen-Bio (Chengdu, China). CD44 monoclonal antibody was purchased from Elabscience Biotechnology (Wuhan, China). Glyceraldehyde-3-phosphate dehydrogenase (GAPDH) antibody and Ki67 antibody were purchased from Biosen (Shanghai, China).

### Cell lines

Human SCLC cell lines, including NCI-H446, NCI-H69AR, and NCI-H69, were obtained from the American Type Culture Collection. H446CDDP (the chemoresistant subline) was established by exposing H446 cells to gradually increasing concentrations of CDDP (up to 0.5 μg/ml) over 12 months. H69AR cells were grown in RPMI 1640 enriched with 20% FBS, while H446 and H69 cells were grown in RPMI 1640 medium containing 10% FBS. H446CDDP cells were grown in RPMI 1640 medium supplemented with 10% FBS (Gibco, Waltham, WA, USA) and 0.5 μg/ml of CDDP. Prior to any experiments, H446CDDP and H69AR (the chemoresistant sublines) were placed in drug-free medium and allowed to acclimate for at least 2 weeks. All cell lines were kept in a humidified incubator at 37 °C with 5% CO_2_ to prevent contamination by mycoplasma.

### RNA sequencing and bioinformatic analysis

Total RNA was isolated from H69 and H69AR cells and subjected to transcriptome sequencing analysis. Differential gene expression analysis identified genes with significant expression changes [|Log_2_FC| > 0.585, *P* < 0.05, false discovery rate (FDR) < 0.05]. Gene expression data for PRMT1 were obtained from the Gene Expression Omnibus (GEO) database (GSE30219) [53]. Clinical information associated with this dataset was used for survival analysis. mRNA stability was calculated using optimal components linear regression (OCLR) to compare the differences between the PRMT1 high and PRMT1 low groups [54]. Single-cell RNA sequencing (scRNA-seq) data from the PRJCA006026 dataset [55], profiling primary tumors and matched adjacent normal tissues from 11 SCLC patients, were analyzed. *t*-distributed stochastic neighbor embedding (t-SNE) was used for dimensionality reduction to visualize cell clusters.

### Synthesis of extended DCLX069-ssDNA

Initially, the ssDNAs extended from the stable strands (A16, A45, B16, B45, C16, C45) and functionalized with a carboxyl group (COOH) at the 5′ end were reacted with EDC at a ratio of 1:5 for 10 min. Following this, the mixture was centrifuged 3 times using Microcon centrifugal filtration devices (3 kDa molecular weight cutoff filters) at 15,000 rpm for 10 min (Millipore) to eliminate any residual EDC. Next, NHS was introduced and allowed to react for 40 min. The solution was then subjected to 3 additional centrifugation cycles using Microcon centrifugal filtration devices (3 kDa molecular weight cutoff filters) at 15,000 rpm for 10 min (Millipore) to remove any leftover NHS. Finally, the PRMT1 inhibitor DCLX069 (50 μl, 100 μM) was added and allowed to react for 4 h. Afterward, DCLX069-ssDNA was purified via centrifugation using Microcon centrifugal filtration devices (3 kDa molecular weight cutoff filters) at 15,000 rpm for 10 min (Millipore) to remove any residual DCLX069.

### Assembly of the nontargeted triangle, targeted triangle, nontargeted DNM, and DNM

Four distinct DNA origami products (including nontargeted triangle, targeted triangle, nontargeted DNM, and DNM) were assembled in this study.

For the nontargeted triangle group (5 nM), the following components were combined in a 200-μl PCR tube: 10× TAE-Mg^2+^ buffer (10 μl, pH 8.0, comprising 200 mM HAc, 125 mM MgAc·4H_2_O, 20 mM EDTA-NH_2_, and 400 mM tris), Milli-Q water (65 μl), stable strands (250 nM each, 20 μl, as listed in Table S1), and M13mp18 ssDNA (100 nM, 5 μl). For the targeted triangle group (5 nM), the following components were combined in a 200-μl PCR tube: 10× TAE-Mg^2+^ buffer (10 μl, pH 8.0, comprising 200 mM HAc, 125 mM MgAc·4H_2_O, 20 mM EDTA-NH_2_, and 400 mM tris), Milli-Q water (65 μl), stable strands (which included CD44 aptamer strands, 1 μM each, 20 μl, as detailed in Table S2), and M13mp18 ssDNA (100 nM, 5 μl). For the nontargeted DNM group (5 nM), the following components were combined in a 200-μl PCR tube: 10× TAE-Mg^2+^ buffer (10 μl, pH 8.0, comprising 200 mM HAc, 125 mM MgAc·4H_2_O, 20 mM EDTA-NH_2_, and 400 mM tris), Milli-Q water (65 μl), stable strands (which included DCLX069-ssDNA, 1 μM each, 20 μl, as detailed in Table S3), and M13mp18 ssDNA (100 nM, 5 μl). For the DNM group (5 nM), the following components were combined in a 200-μl PCR tube: 10× TAE-Mg^2+^ buffer (10 μl, pH 8.0, comprising 200 mM HAc, 125 mM MgAc·4H_2_O, 20 mM EDTA-NH_2_, and 400 mM tris), Milli-Q water (65 μl), stable strands (which included CD44 aptamer strands and DCLX069-ssDNA, 1 μM each, 20 μl, as detailed in Table S4), and M13mp18 ssDNA (100 nM, 5 μl). The mixture was then subjected to a standard thermal incubation protocol (95 °C for 3 min, followed by a cooling phase from 95 °C to 15 °C at a rate of 0.1 °C every 10 s) using a PCR thermal cycler (ABI, PCR System 9700) [56,57].

Then, the mixture was subjected to a standard thermal incubation procedure (95 °C for 3 min, followed by a cooling process from 95 °C to 15 °C at a rate of 0.1 °C every 10 s) using a PCR thermal cycler (ABI, PCR System 9700). These prepared DNA origami structures were purified 3 times with Microcon centrifugal filtration devices (100 kDa molecular weight cutoff filters, Millipore) to remove the excess staple strands. Each sample was diluted to 100 μl for further use.

### Preparation of targeted triangle-CDDP and DNM-CDDP

The targeted triangle and DNM were separately cultured with superfluous CDDP at 37 °C for about 3 to 4 h to form targeted triangle-CDDP and DNM-CDDP. After incubation, the nanotriangle-CDDP and DNM-CDDP were purified 3 times with Microcon centrifugal filtration devices (100 kDa molecular weight cutoff filters, Millipore) to remove the excess CDDP.

### AFM characterization

An atomic force microscope (Bruker, Multimode Nanoscope VIII) was employed to examine the morphology and dimensions of the synthesized DNA origami products. Prior to imaging, the samples (1 nM, 8 μl) were adsorbed onto the mica surface for 3 to 5 min. The operation was performed in liquid under Peak force mode with a SCANASYST-FLUID+ tip (Bruker). NanoScope Analysis 1.8 (Bruker) was utilized to analyze the collected images.

### Drug release efficiency of DNM-CDDP

DNM-CDDP samples (10 nM, 2 ml) were loaded into dialysis bags (3 kDa) and dialyzed against tumor tissue lysate for 0, 0.083, 0.17, 0.25, 0.5, 0.75, 1, 1.5, 2, 3, 4, 6, 8, 10, 12, 16, 20, 24, 30, and 36 h at 37 °C. With continuous shaking, the dialysate was removed at each point in time for the following tests.

To assess the relative efficiency of DCLX069 release, the cumulative amount of released DCLX069 was quantified by detecting the absorbance (230 nm) using UV–Vis spectroscopy. Subsequently, the DCLX069 release efficiency was determined through the following equation: DCLX069 release efficiency = (cumulative DCLX069 released/DCLX069 loaded) × 100%.

To determine the relative efficiency of CDDP release, the cumulative amount of released CDDP was quantified using ICP-MS measurements. Subsequently, the CDDP release efficiency was determined through the following equation: CDDP release efficiency = (cumulative CDDP released/CDDP loaded) × 100%.

### Agarose gel electrophoresis analysis and fluorescence imaging

We executed the agarose gel electrophoresis assays via agarose gel (1%). The experiments were carried out in ice-cold 1× TAE-Mg^2+^ buffer (40 mM tris, 2 mM EDTA-NH_2_, 12.5 mM MgAc·4H_2_O, and 20 mM HAc, pH 8.0), while the run time was 1 h and the run voltage was 100 V. A chemiluminescence imaging system (Bio-Rad, ChemiDoc MP) was used to image the gel red-stained gel bands and samples labeled with specific fluorescence. Agarose gel electrophoresis and fluorescence imaging were used to analyze the synthesis process of DNM and the cargo release process.

For analyzing the synthetic process of DNM, the DNA origami samples were labeled with specific fluorophores. The DNA origami framework was labeled with Cy5, while the CD44 aptamer was labeled with Cy7 and the DCLX069-ssDNA was labeled with Alexa Fluor 488. Next, an aliquot of 24 μl of M13 DNA sample (20 μl of M13mp18 ssDNA, 2 nM; 4 μl of 6× loading buffer) was loaded into the first lane of 1% agarose gel as the marker, while the DNA origami samples (24 μl each, containing 20 μl of DNA origami product, 4 μl of 6× loading buffer) were loaded into the other lanes. The lane assignments were as follows: (a) M13 mp18 ssDNA, (b) nontargeted triangle, (c) targeted triangle, (d) nontargeted DNM, and (e) DNM. The excitation wavelengths for fluorescence imaging were as follows: 300 nm for GelRed (gray), 645 nm for Cy5-labeled DNA origami framework (red), 750 nm for Cy7-labeled CD44 aptamers (blue), and 488 nm for Alexa Fluor 488-labeled DCLX069-ssDNA (green).

For analyzing the cargo release from DNM, the DNA origami framework was labeled with Cy5 and ssDNA was labeled with Alexa Fluor 488. Next, the DNM samples (10 nM, 2 ml) were incubated with tumor tissue lysate for 0.5, 1, 4, 6, 8, 12, and 24 h at 37 °C. After incubation, the samples (24 μl each, containing 20 μl of DNM samples, and 4 μl of 6× loading buffer) were loaded into the lanes of 1% agarose gel. After agarose gel electrophoresis, fluorescence imaging was executed. The excitation wavelengths for fluorescence imaging were as follows: 645 nm for Cy5-labeled DNA origami framework (red) and 488 nm for Alexa Fluor 488-labeled ssDNA (green).

### Fluorescence colocalization imaging analysis

Fluorescence colocalization imaging analysis was employed to analyze the synthesis process of DNM and the cargo release process.

For analyzing the synthetic process of DNM, the DNA origami framework was labeled with Cy5, while the CD44 aptamers were labeled with Cy7, and the DCLX069-ssDNA was labeled with Alexa Fluor 488. An imaging system (Bio-Rad, ChemiDoc MP) was used to image the DNM samples (10 nM, 2 ml) labeled with the above fluorescence. The excitation wavelengths for fluorescence imaging were as follows: 645 nm for Cy5-labeled DNA origami framework (red), 750 nm for Cy7-labeled CD44 aptamers (blue), and 488 nm for Alexa Fluor 488-labeled DCLX069-ssDNA (green).

For analyzing the cargo release from DNM, the DNA origami framework was labeled with Cy5, and ssDNA was labeled with Alexa Fluor 488. Subsequently, DNM samples (10 nM, 2 ml) were incubated with tumor tissue lysate for durations of 0, 4, 12, and 24 h at 37 °C. Following this incubation, the samples were captured using the ChemiDoc MP imaging system. The excitation wavelengths for fluorescence imaging were as follows: 645 nm for Cy5-labeled DNA origami framework (red) and 488 nm for Alexa Fluor 488-labeled ssDNA (green).

### EDS detection

EDS was used for detecting elemental platinum (Pt) and elemental phosphorus (P) elements in DNM and DNM-CDDP groups. EDS data were obtained with a 1040 Oxford Instruments EDX detector. Images were obtained using the secondary electron detector at an accelerating voltage of 5 kV.

### Western blot

In this study, Western blotting was employed to assess the expression levels of PRMT1 and SOX2 among SCLC sublines, examine the SOX2 expression following treatment with DCLX069, and evaluate the stemness-reducing effect of DNM-CDDP.

To assess PRMT1 and SOX2 expression in SCLC sublines, protein extracts were obtained from H69, H69AR, H446, and H446CDDP cells and quantified using protein extraction and bicinchoninic acid (BCA) protein assay kits (CWbio, Jiangsu, China). Cell lysates combined with sodium dodecyl sulfate–polyacrylamide gel electrophoresis (SDS-PAGE) loading buffer (Beyotime Biotechnology, Shanghai, China) were heated to 100 °C for 10 min. The protein samples were then subjected to electrophoresis in SDS-PAGE gels before being transferred to polyvinylidene difluoride membranes (Merck Millipore, Billerica, MA, USA). The membranes were incubated overnight at 4 °C with primary antibodies against PRMT1 or SOX2 after being blocked with 5% bovine serum albumin. Following 3 washes with tris-buffered saline containing Tween, secondary antibodies were introduced to minimize nonspecific binding. Chemiluminescent signals were subsequently detected and recorded digitally using a Bio-Rad imaging system (Bio-Rad, Hercules, CA, USA).

To assess SOX2 expression after DCLX069 treatment and to evaluate the stemness-reducing effects of DNM-CDDP, H69AR cells were initially seeded in 6-well plates and incubated overnight. The cells were subsequently treated with either PBS and DCLX069 or PBS, targeted triangle, CDDP, targeted triangle-CDDP, DNM, and DNM-CDDP in 1 ml of complete RPMI 1640 culture medium for 12 or 24 h. Following the incubation, protein extraction, quantification, and electrophoresis were performed as above-described.

### CCK-8 assay

The CCK-8 assay was used for evaluating the cytotoxic effects of combining CDDP with DCLX069, as well as the chemoresistance-reversing effect of DNM-CDDP on SCLC in vitro.

To evaluate the cytotoxic effects of the combination of CDDP and DCLX069 on drug-resistant SCLC sublines, H69AR cells (ranging from 5,000 to 20,000 cells per well) were seeded into a 96-well plate containing complete medium. The cells were subsequently treated with a range of concentrations of the following compounds: (a) DCLX069 (0, 0.5, 1, 1.5, 2, 2.5, 3 μM) for 24 h, (b) CDDP (0, 25, 50, 75, 100, 125, 150 μM) for 24 h, (c) cotreatment with DCLX069 (0, 0.5, 1, 1.5, 2, 2.5, 3 μM) and CDDP (0, 25, 50, 75, 100, 125, 150 μM) for 24 h, and (d) pretreatment with DCLX069 (0, 0.5, 1, 1.5, 2, 2.5, 3 μM) for 24 h, followed by cotreatment with CDDP (0, 25, 50, 75, 100, 125, 150 μM) for an additional 24 h. Optical density was recorded at 450 nm using a microplate reader (Biotek, Winooski, VT, USA). Cell viability (%) was calculated as follows:

$$\text{Cell viability}\;\left(\%\right)=\frac{\mathrm{OD}\left(\text{treatment}\right)-\mathrm{OD}\left(\text{blank}\right)}{\mathrm{OD}\left(\mathrm{PBS}\right)-\mathrm{OD}\left(\text{blank}\right)\;}\times 100\%$$

To evaluate the chemoresistance-reversing effect of DNM-CDDP on SCLC in vitro, 5,000 to 20,000 H69AR cells were plated into each well of a 96-well plate containing complete medium. The cells were subsequently treated with gradient concentrations of the following agents: PBS, targeted triangle, CDDP, targeted triangle-CDDP, DNM, and DNM-CDDP (corresponding to Log CDDP concentrations of 0, 0.5, 1, 1.5, and 2 μg/ml) for a duration of 24 h. Optical density was measured at 450 nm using an absorbance microplate reader, and cell viability (%) was assessed as described above.

### Live-dead viability assay

H69AR cells were plated in a 6-well plate at a concentration of 1 × 10^6^ cells per well at 37 °C. After 24 h of incubation, the cells were divided into 6 groups and treated with PBS, targeted triangle, CDDP, targeted triangle-CDDP, DNM, and DNM-CDDP (corresponding to Log CDDP: 2 μg/ml) for 24 h. Then, the cells were harvested. Dye Reagent 1 and Dye Reagent 2 were mixed in equal volumes to form Mixed Dye Reagent, and then 25 μl of the cell suspension was aspirated by pipette and mixed gently with 1 μl of Mixed Dye Reagent. Ten microliters of the above mixture was placed on a clean slide, and the cells were covered with a coverslip. Finally, fluorescence images were observed with a Nikon confocal laser microscope (AX NIS-Elements 5.4, Japan).

### Confocal imaging analysis

Confocal imaging analysis was employed to investigate the cellular drug release profile, as well as the tumor-targeting ability of DNM between H69 and H69AR cells, the tumor-targeting ability of DNM and nontargeted DNM, and the relationship of the tumor-targeting ability of DNM and prolonged incubation time.

To investigate cellular drug release, the DNA origami framework was first labeled with Cy5, and the ssDNA was tagged with Alexa Fluor 488. Subsequently, H69AR and H446CDDP cells were cultured overnight at 37 °C in 35 mm confocal dishes. Afterward, the cells were washed twice with PBS and treated with DNM-CDDP at a final concentration of 5 nM in 1 ml of RPMI 1640 complete culture medium at 37 °C for 0.5 and 6 h. Following treatment, the cells were rinsed 3 times with PBS solution and fixed using 4% paraformaldehyde. The nuclei were then stained with 1 μg/ml DAPI for 5 min. Finally, fluorescence imaging was performed using laser confocal microscopy (Nikon, AX NIS-Elements 5.4, Japan). Excitation wavelengths were 405 nm for DAPI, 488 nm for Alexa Fluor 488, and 640 nm for Cy5.

To evaluate the tumor-targeting capability of DNM in H69 and H69AR cells, the cells were initially seeded in 35-mm confocal dishes and cultured overnight at 37 °C. Afterward, the cells were rinsed twice with PBS and treated with Cy5-labeled DNM (5 nM) in 1 ml of culture medium for 6 h at 37 °C. After incubation, the cells were washed thrice with PBS and fixed with 4% paraformaldehyde. The cytoskeleton was stained for 10 min with 1 μg/ml phalloidin, and the nuclei were stained with 1 μg/ml DAPI for 5 min. Finally, fluorescence imaging was performed as above-described.

To further assess the tumor-targeting capabilities of DNM and nontargeted DNM, H69AR cells were treated similarly. After being seeded in confocal dishes and cultured overnight at 37 °C, the cells were rinsed with PBS and treated with either DNM or nontargeted DNM (both labeled with Cy5) at a 5 nM concentration in 1 ml of RPMI 1640 complete culture medium for 6 h at 37 °C. The imaging procedure was conducted as above-described.

Next, to explore the relationship between the tumor-targeting capacity of DNM and extended incubation times, H69AR cells were seeded in 35-mm confocal dishes and cultured overnight at 37 °C. After rinsing twice with PBS, the cells were incubated with DNM labeled with Cy5 at 5 nM in 1 ml of RPMI 1640 complete culture medium for durations of 1, 3, and 6 h at 37 °C, followed by imaging as above-described.

### Flow cytometry analysis

Flow cytometry analysis in this study was utilized for detecting the CD44 expression between H69 and H69AR cells, the tumor-targeting ability of DNM between H69 and H69AR cells, the tumor-targeting ability of DNM and nontargeted DNM, the relationship of the tumor-targeting ability of DNM and prolonged incubation time, and in vitro anticancer effect of DNM-CDDP through cell apoptosis assay.

To determine CD44 expression differences between H69 and H69AR cells, 0.4 × 10^6^ cells were plated in 6-well plates and incubated overnight at 37 °C. The cells were then collected and reacted with 5 μl of CD44 antibody in 100 μl of PBS for 30 min. After reaction, the cells were rinsed 3 times with PBS buffer to remove unbound antibody. Subsequently, the intensity of cell fluorescence was analyzed by flow cytometry (Beckman Cytoflex S) using the phycoerythrin (PE) channel.

To evaluate the targeting capability of DNM, the tumor cells were cultured overnight at 37 °C in 6-well plates. Following this, they were incubated with Cy5-labeled DNM or nontargeted DNM, with a final concentration of 5 nM triangle DNA origami in 1 ml of RPMI 1640 complete culture medium for durations of 1, 3, or 6 h. After the drug treatment, the cells were enzymatically dissociated for 3 min to obtain a cell suspension, which was then washed 3 times with PBS. Subsequently, Cy5 fluorescence was evaluated using a flow cytometry system operating with the allophycocyanin (APC) channel.

For the cell apoptosis assay, a total of 1 × 10^6^ H69AR cells were plated in 2 ml of culture medium and allowed to incubate for 24 h in a 6-well plate. Then, the cells were divided into 6 groups (respectively treated with PBS, targeted triangle, CDDP, targeted triangle-DDP, DNM, and DNM-CDDP for 24 h). Then, the cells were stained with 5 μl of annexin V-FITC for 15 min and 10 μl of PI for 5 min at room temperature. After that, the fluorescence intensity of cells was detected by a flow cytometer with both PE and FITC channels.

### H69AR tumor-bearing mice model and tumor xenograft experiments

All animal-related protocols were approved by the Institutional Animal Care and Use Committee (IACUC) of Zhujiang Hospital, Southern Medical University (LAEC-2022-176), following institutional guidelines for animal welfare and usage. Specifically, male BALB/c nude mice, 4 to 6 weeks old, were sourced from the Guangdong Medical Laboratory Animal Center. Mice were housed under standard conditions with a 12-h light/dark cycle, controlled temperature (22 ± 2 °C), and humidity (50% to 60%). Animals had free access to food and water, and environmental enrichment, including nesting material and shelters, was provided. All procedures were performed in accordance with institutional guidelines and approved by the IACUC of Zhujiang Hospital, Southern Medical University, ensuring compliance with ethical standards and reproducibility. The BALB/c nude mice were allowed a 1-week acclimatization period before being injected subcutaneously with approximately 5 × 10^6^ H69AR cells in the right lower flank. Euthanasia was performed via CO_2_ asphyxiation, followed by cervical dislocation.

### Biodistribution analysis

H69AR tumor-bearing mice were randomly assigned to 3 groups, with 3 mice in each group. They received identical doses of either free Cy5, Cy5-labeled nontargeted DNM, or Cy5-labeled DNM, calculated based on the concentration of Cy5. The treatments were administered via tail vein injection in a volume of 100 μl. Fluorescence imaging was conducted using an animal imaging system (IVIS Spectrum, Living Image 4.5.2) at 1, 4, 12, 24, and 36 h post-injection. At 1, 4, 12, and 36 h after administration, the mice were euthanized to collect tumor tissues and major organs for ex vivo fluorescence imaging. The excised tumors were subjected to immunofluorescence staining with DAPI. Then, fluorescence intensity of Cy5 of the tumor slices was observed by the Nikon confocal laser microscope (AX NIS-Elements 5.4, Japan) to detect the targeted ability of free Cy5, nontargeted DNM, and DNM.

### Evaluation of in vivo antitumor efficacy

When the tumor volume in H69AR mice reached 80 mm^3^, the mice were randomly allocated into 6 groups, each consisting of 6 mice. The treatments administered through tail vein injection included 0.9% saline, targeted triangle, CDDP, targeted triangle-CDDP, DNM, and DNM-CDDP, delivered in a volume of 100 μl every 8 d across 3 treatment cycles. The CDDP content in the CDDP, targeted triangle-CDDP, and DNM-CDDP groups was administered at equivalent doses (CDDP: 3 mg/kg). The size of the xenografts was assessed every 3 d throughout the 24-d treatment period. Tumor dimensions were recorded using a digital vernier caliper, measuring both the longest (*a*) and shortest (*b*) diameters, with volume calculated using the formula *V* = 0.5*ab*^2^. Additionally, the mouse’s weight was recorded every other day using an electronic balance during the entire treatment duration. At the end of the 24-d period, the mice were euthanized, and tumor tissues were photographed, weighed, and processed for H&E staining, IHC staining (caspase-3, γ-H2AX, Ki67), and immunofluorescence staining (TUNEL). For the evaluation of survival, the survival periods of the mice treated with different formulations were recorded during treatment.

### Biosafety investigation

Biosafety investigation included cytokine level detection by enzyme-linked immunosorbent assay (ELISA) assay, routine blood test, hepatic and renal function-related biomarker test, and histopathological evaluations of the major organs.

For the detection of cytokine levels using the ELISA assay, 6- to 8-week-old male C57BL/6 mice were randomly allocated into 6 groups (*n* = 3 per group). The mice received intravenous injections of 0.9% saline, targeted triangle, CDDP, targeted triangle-CDDP, DNM, and DNM-CDDP, all administered in a 100-μl injection volume. Blood samples were collected after 6 h. Subsequently, the levels of TNF-α, IFN-α, and IL-6 were evaluated using specific ELISA kits (Abcam, USA), with 3 independent measurements obtained from each cytokine, corresponding to 3 mice.

For routine blood analyses and tests related to hepatic and renal function biomarkers, BALB/c nude mice aged 6 to 8 weeks were assigned to 3 groups, each consisting of 3 mice. The mice received intravenous injections of 0.9% saline, CDDP, and DNM-CDDP, all administered in a 100-μl injection volume. After 24-d treatment, murine whole blood was collected for routine blood tests and hepatic and renal function-related biomarker tests before sacrifice.

For histopathological assessments of the major organs, BALB/c nude mice aged 6 to 8 weeks were allocated into 3 groups, each consisting of 3 mice. The mice received intravenous injections of 0.9% saline, CDDP, and DNM-CDDP, all administered in a 100-μl injection volume. After 24-d treatment, the major organs (hearts, livers, spleens, lungs, and kidneys) were harvested and stained with H&E staining for the histopathological evaluations.

### Statistical analysis

All the results are presented as the mean ± SD (*n* ≥ 3). One-way analysis of variance (ANOVA) with Tukey’s post hoc test was used for statistical comparisons among multiple groups (more than two). Two-sided log-rank (Mantel–Cox) test was used for the statistical comparison of the survival study. Statistical analysis was conducted using Prism 8.0 (GraphPad, San Diego, CA, USA). Statistical analysis symbols were as follows: ns = not significant, *P < 0.05, **P < 0.01, ***P < 0.001, ****P < 0.0001.

## Data Availability

All data generated or analyzed during this study are included in this current article and its additional files.
