# Supplementary material for: A DNA Nanomachine Modulates the Stemness-Associated Signaling Pathways for Overcoming Chemoresistance by Temporally Programming Drug Release
Source: Research (Wash D C). 2025 Dec 9;8:0999. doi: 10.34133/research.0999 (PMC12687658; doi:10.34133/research.0999)
Supplement: Supplementary 1 — Figs. S1 to S15 Tables S1 to S4 [file research.0999.f1.docx]

Supporting Information

**A DNA nanomachine modulates the stemness-associated signaling pathways for overcoming chemoresistance by temporally programming drug release**

Jie Chen^1, 2, 3†^, Xiaodie Li^1, 4†^, Qian Chen^2†^, Xuyang Zhou^2^, Jialin Zeng^1^, Linlang Guo^2*^, Yinan Zhang^5*^, Dayong Yang^6*^, Chao Zhang^1*^

^1^ Department of Oncology, Zhujiang Hospital, Southern Medical University, Guangzhou, 510282, P. R. China

^2^ *Department of Pathology, Zhujiang Hospital, Southern Medical University Guangzhou, 510282, P. R. China*

^3^ *Department of Radiation Oncology, Cancer Hospital of Shantou University Medical College, Shantou, 515041, P.R. China*

^4^ *Department of Medical Oncology, Sun Yat-Sen University Cancer Center, Guangzhou, 510060, P.R. China*

^5^ *School of Chemical Science and Engineering, Tongji University, Shanghai, 200092, P. R. China*

^6^ *Department of Chemistry, State Key Laboratory of Molecular Engineering of Polymers, Shanghai Key Laboratory of Molecular Catalysis and Innovative Materials, College of Chemistry and Materials, Fudan University, Shanghai, 200438, P. R. China*

** Address correspondence to: Linlang Guo; guoll@smu.edu and Yinan Zhang; 22526@tongji.edu.cn and Dayong Yang; dayongyang@fudan.edu.cn and Chao Zhang; czhangsinap@163.com*

^†^ Jie Chen, Xiaodie Li, Qian Chen contributed equally to this work.


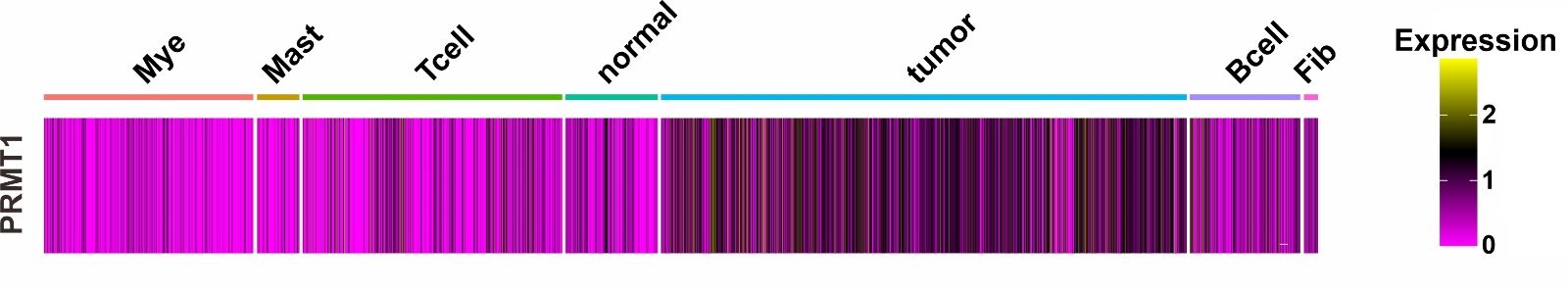


**Figure S1.** Heatmaps of PRMT1 expression in different cell clusters in SCLC.


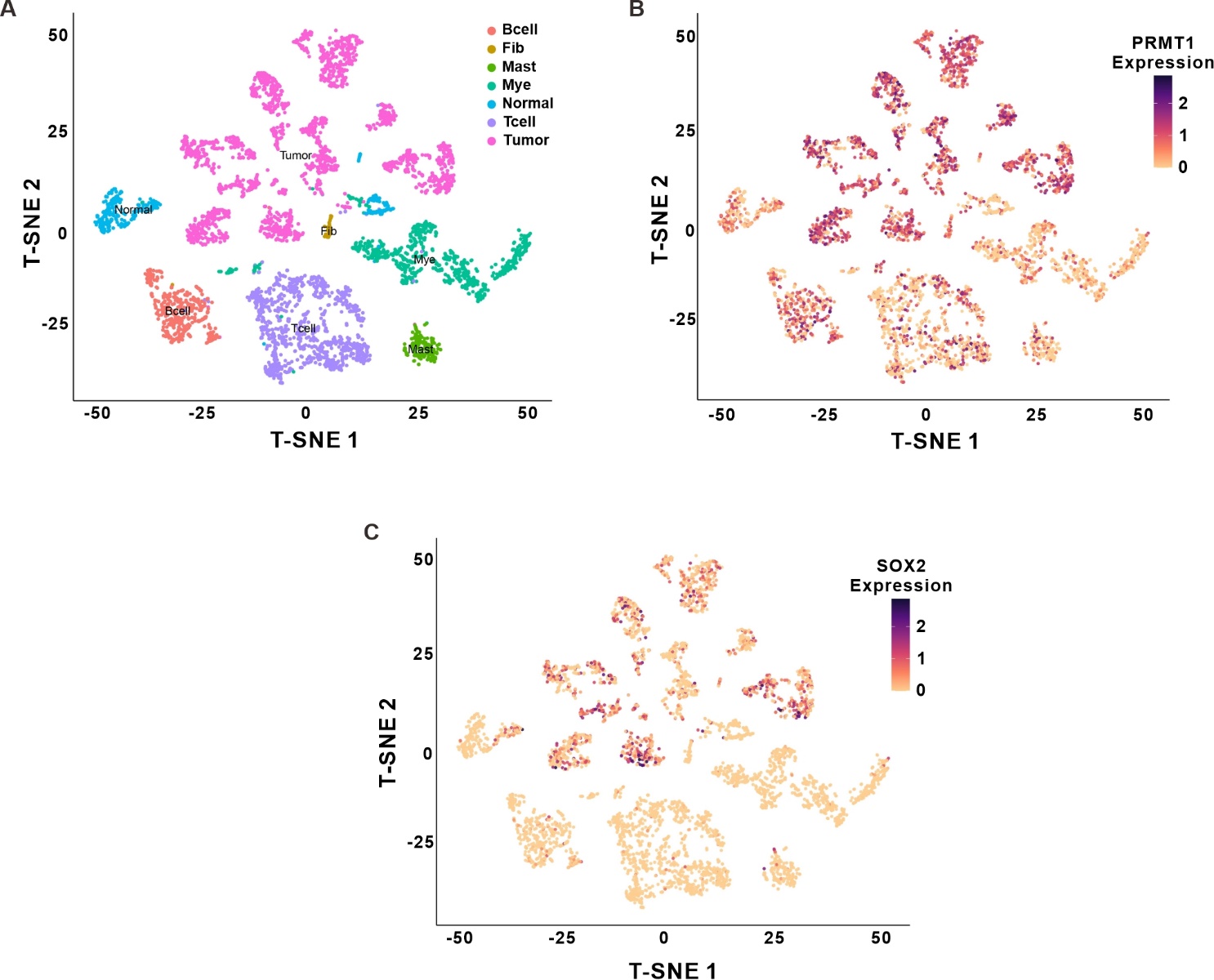
**Figure S2.** Cellular subpopulations and expression of PRMT1 and SOX2 in SCLC. (A) t-SNE visualization of cellular subpopulations. (B) t-SNE visualization of PRMT1 expression across different cell types. (C) t-SNE visualization of SOX2 expression across different cell types.

**
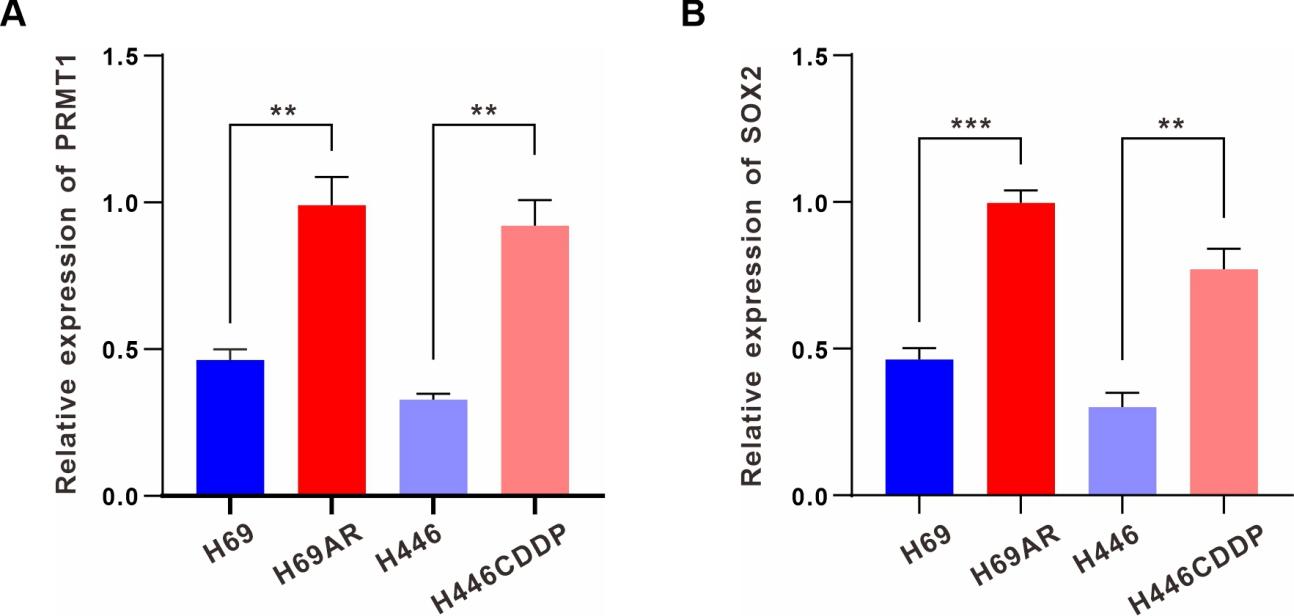
**

**Figure S3.** Quantitative analysis of western blot results. (**A**) PRMT1 expression in drug-resistant (H69AR and H446CDDP) and drug-sensitive (H69 and H446) sublines. (**B**) SOX2 expression in drug-resistant (H69AR and H446CDDP) and drug-sensitive (H69 and H446) sublines. Data are presented as the mean ± SD. *n*=3, ***p* < 0.01, ****p* < 0.001.

**
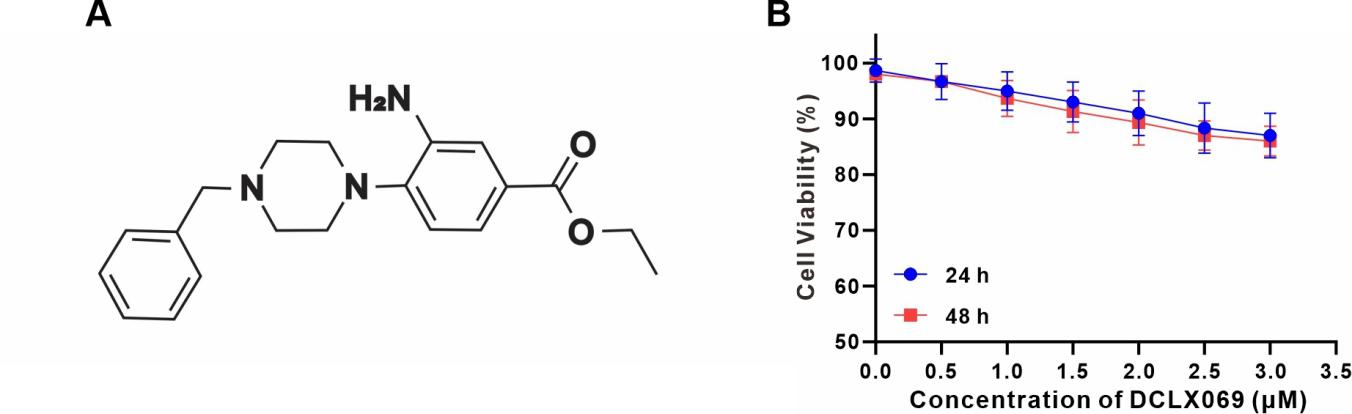
**

**Figure S4.** Structural and functional Investigation of DCLX069. (**A**) Chemical structure of DCLX069. (**B**) Viabilities of H69 AR cells incubated with DCLX069 for 24 hours or 48 hours.


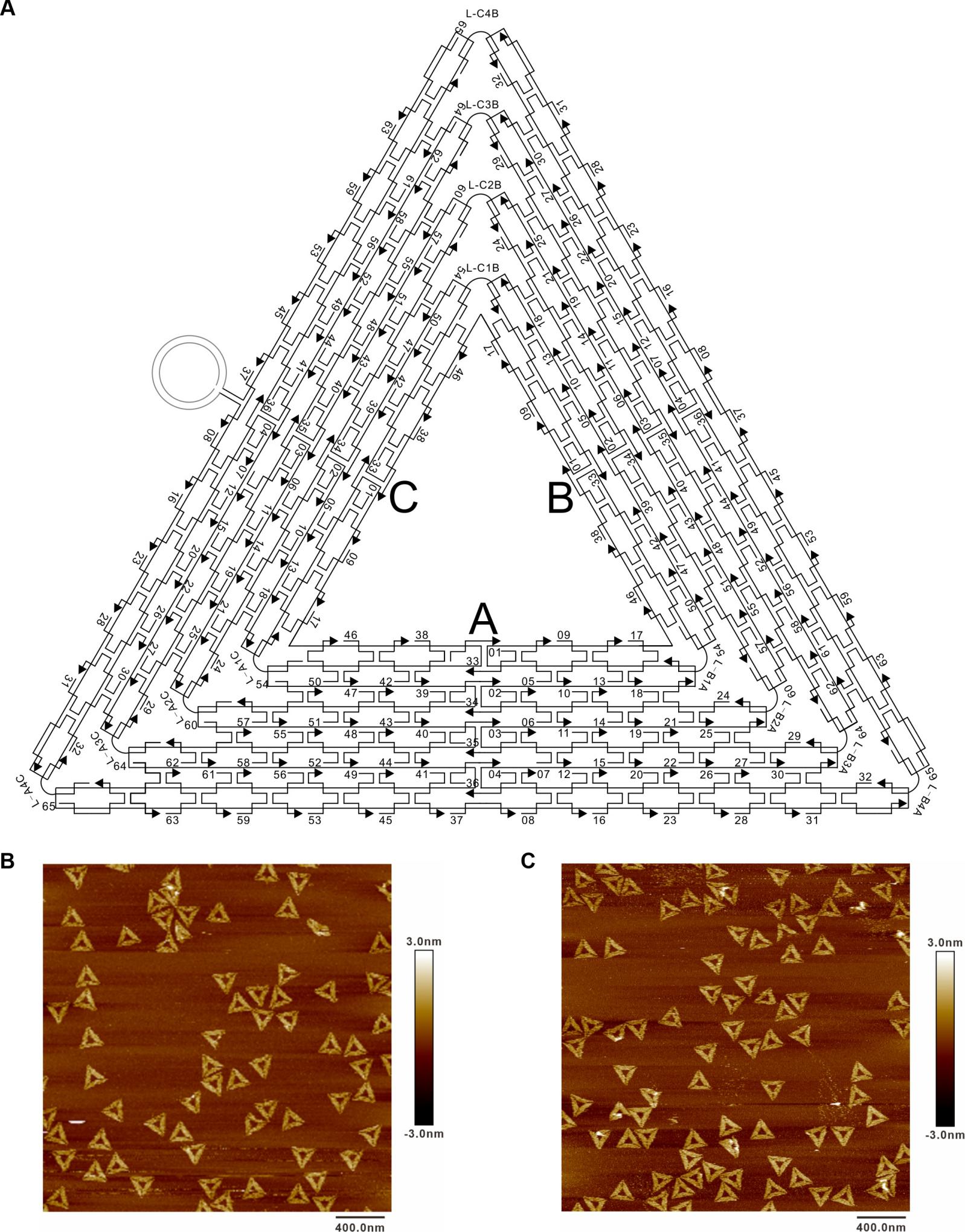


**Figure S5.** Characterization of DNM and DNM-CDDP. (**A**) Design diagram of the triangle DNA origami using caDNAno. (**B**) AFM image of the triangle DNA origami. (**C**) AFM image of the DNM-CDDP.


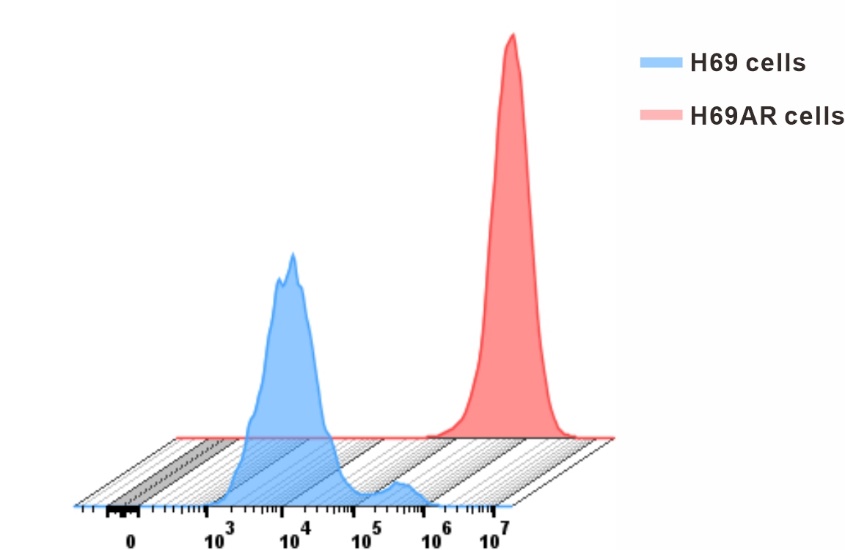


**Figure S6.** Flow cytometry analysis of CD44 expression in H69 and H69AR cells.


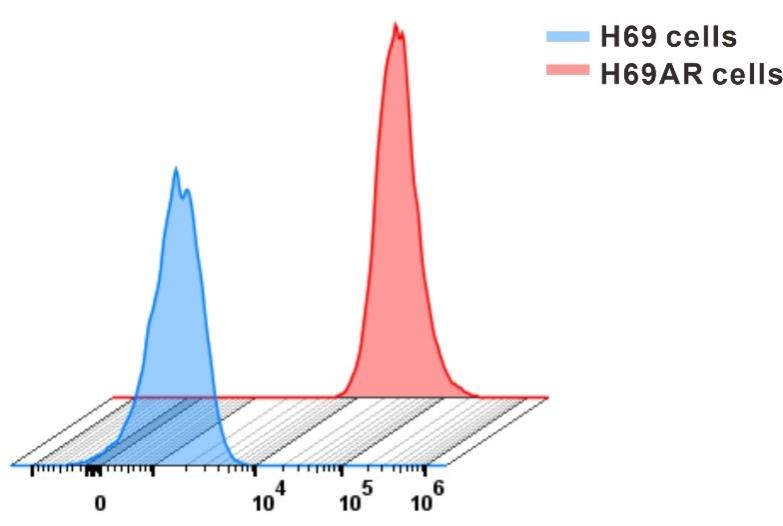


**Figure S7.** Flow cytometry analysis of H69 and H69AR cells treated with Cy5-labeled DNM.


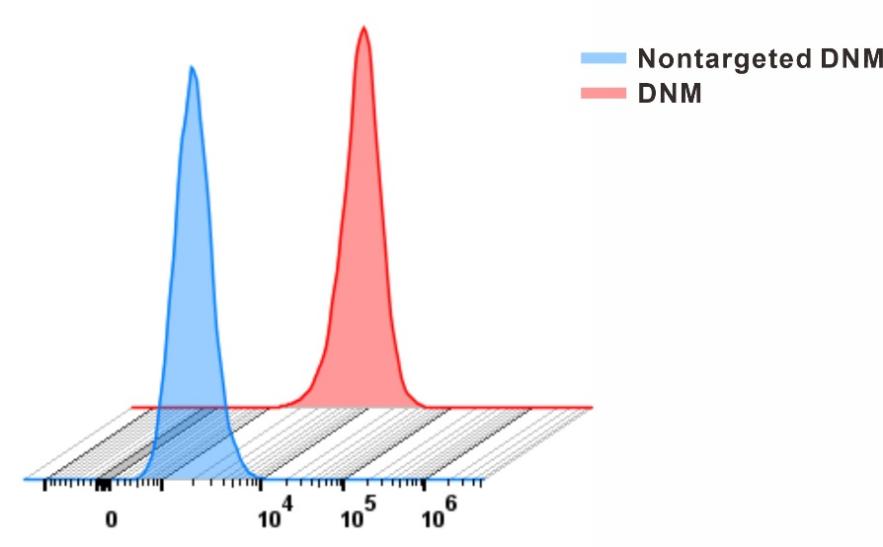


**Figure S8.** Flow cytometry analysis of H69AR cells treated with DNM and non-targeted DNM (both labeled with Cy5).


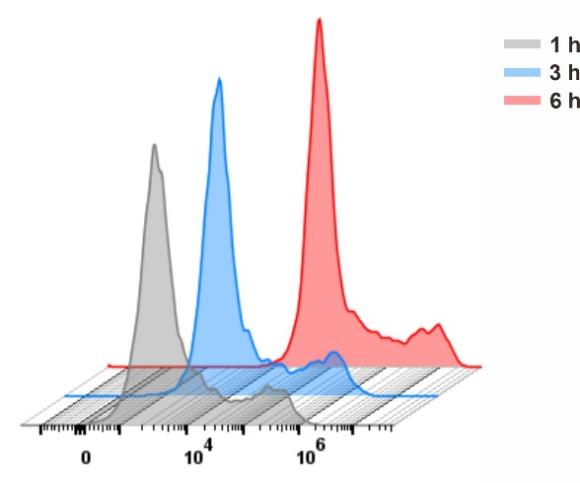


**Figure S9.** Flow cytometry analysis of H69AR cells treated with Cy5-labeled DNM at different time points.


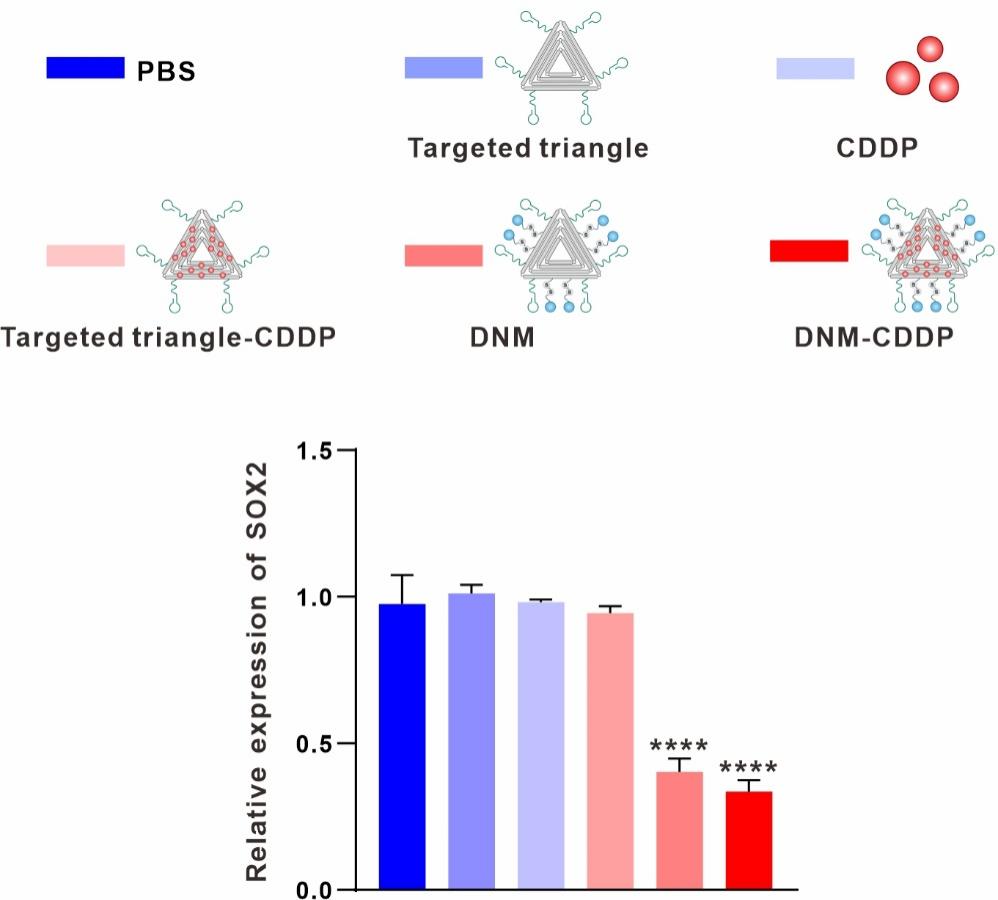


**Figure S10.** SOX2 expression in the indicated treatment groups. Data are presented as the mean ± SD. *n*=3, ****p < 0.0001.


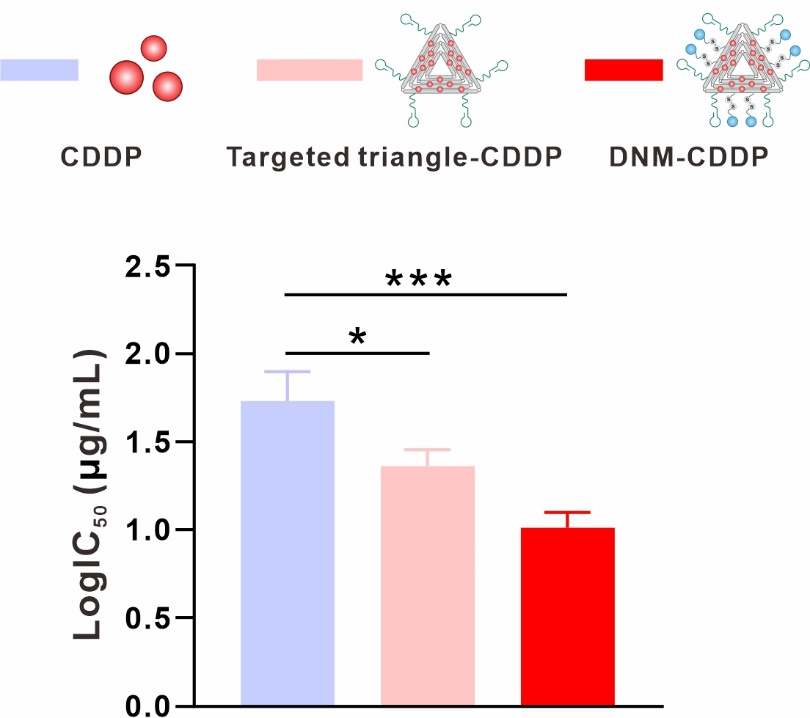


**Figure S11.** LogIC50 value of the indicated treatment groups. Data are presented as the mean ± SD. *n*=3, *p < 0.05, ***p < 0.001.


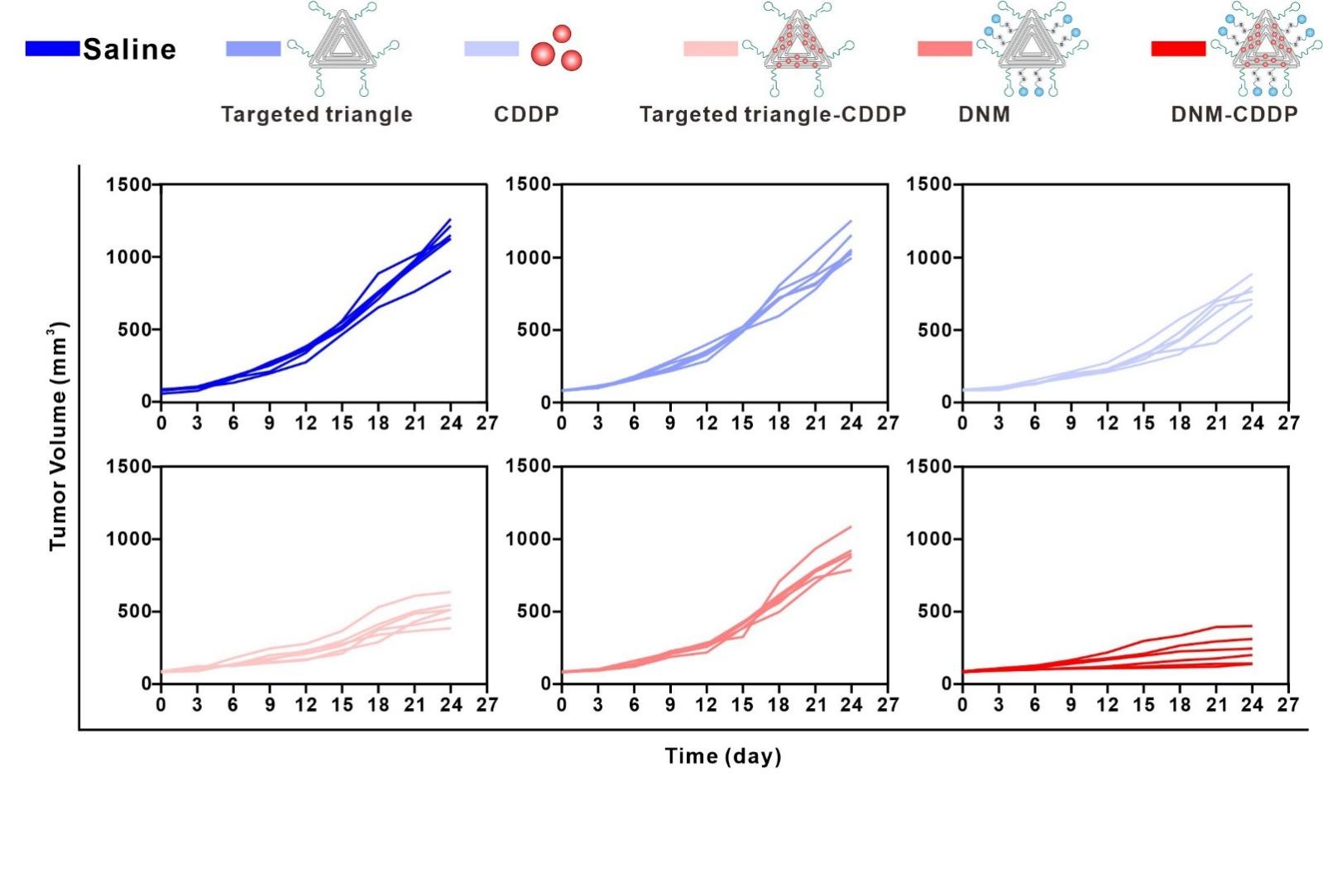
 **Figure S12.** H69AR tumors growth curves of different treatment groups. n=6 for each group.


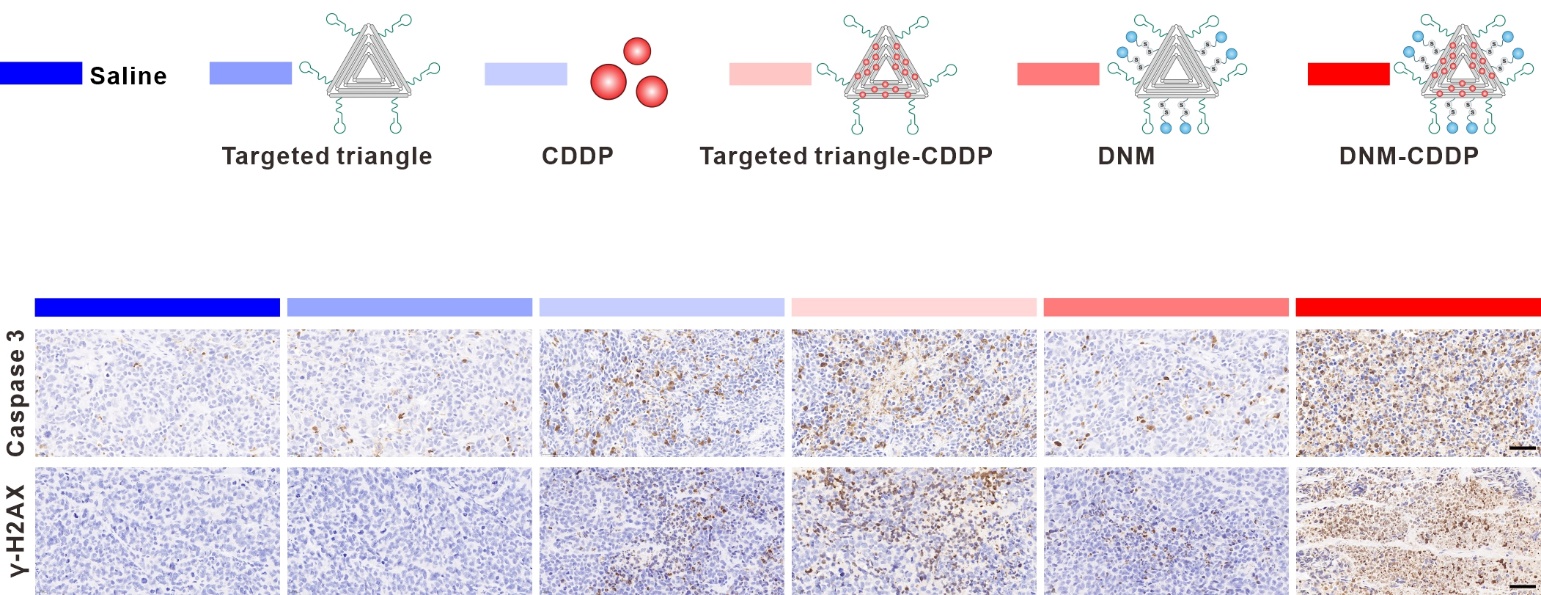


**Figure S13.** Representative images of the tumor sections examined by immunohistochemical staining 24-day post-initiation of indicated treatments. Scale bars, 50 µm.


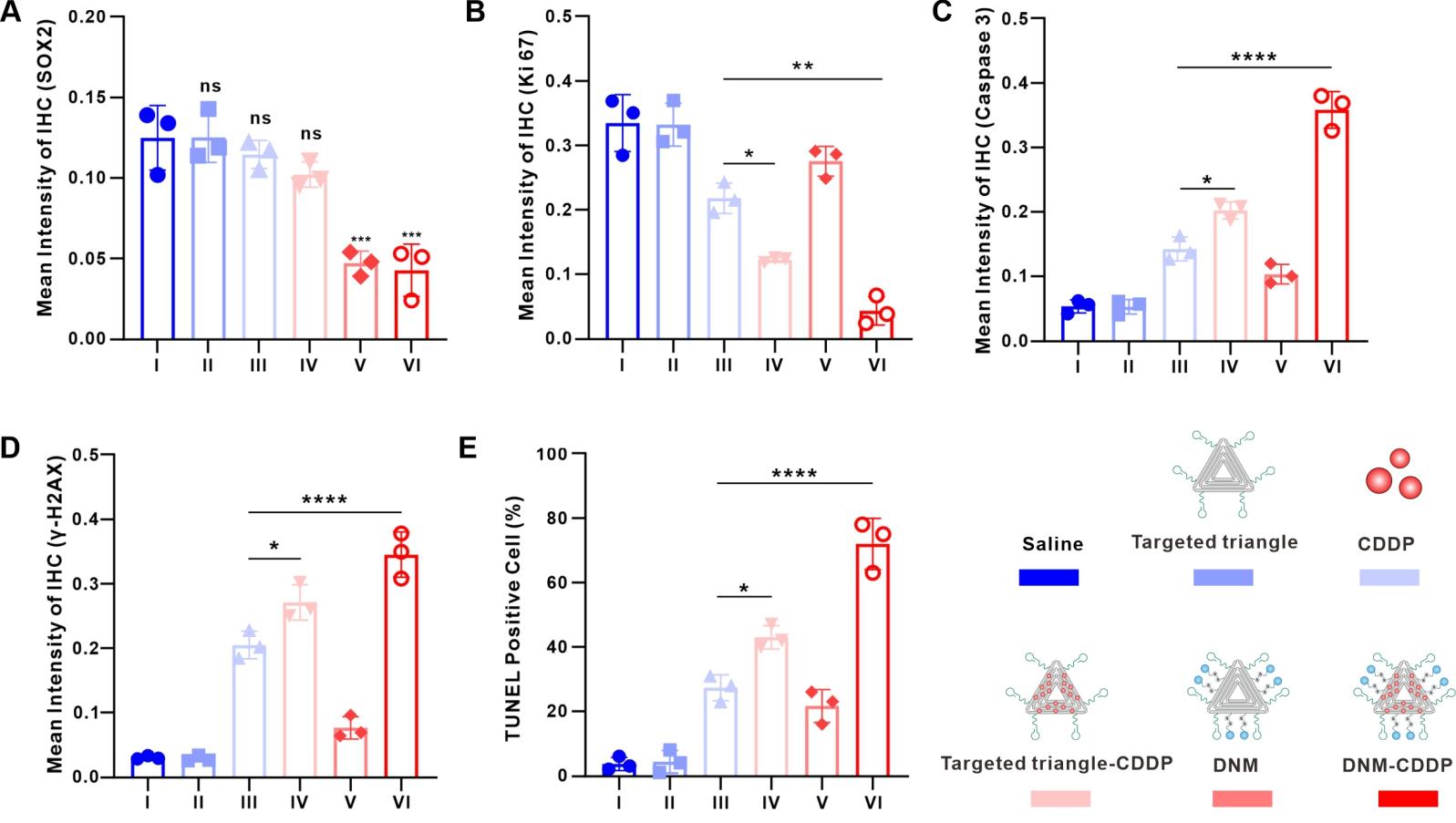


**Figure S14.** Quantitative analysis of immunohistochemical and immunofluorescent staining results. (a) Quantitative analysis of the SOX2 staining area fraction by ImageJ. (b) Quantitative analysis of the Ki-67 staining area fraction by ImageJ. (c) Quantitative analysis of the Caspase 3 staining area fraction by ImageJ. (d) Quantitative analysis of the γ-H2AX staining area fraction by ImageJ. (e) Quantitative analysis of the TUNEL staining area fraction by ImageJ. Data are presented as the mean ± SD. *n*=3, ns: not significant, **p* < 0.05, ***p* < 0.01, ****p* < 0.001, *****p* < 0.0001.

**
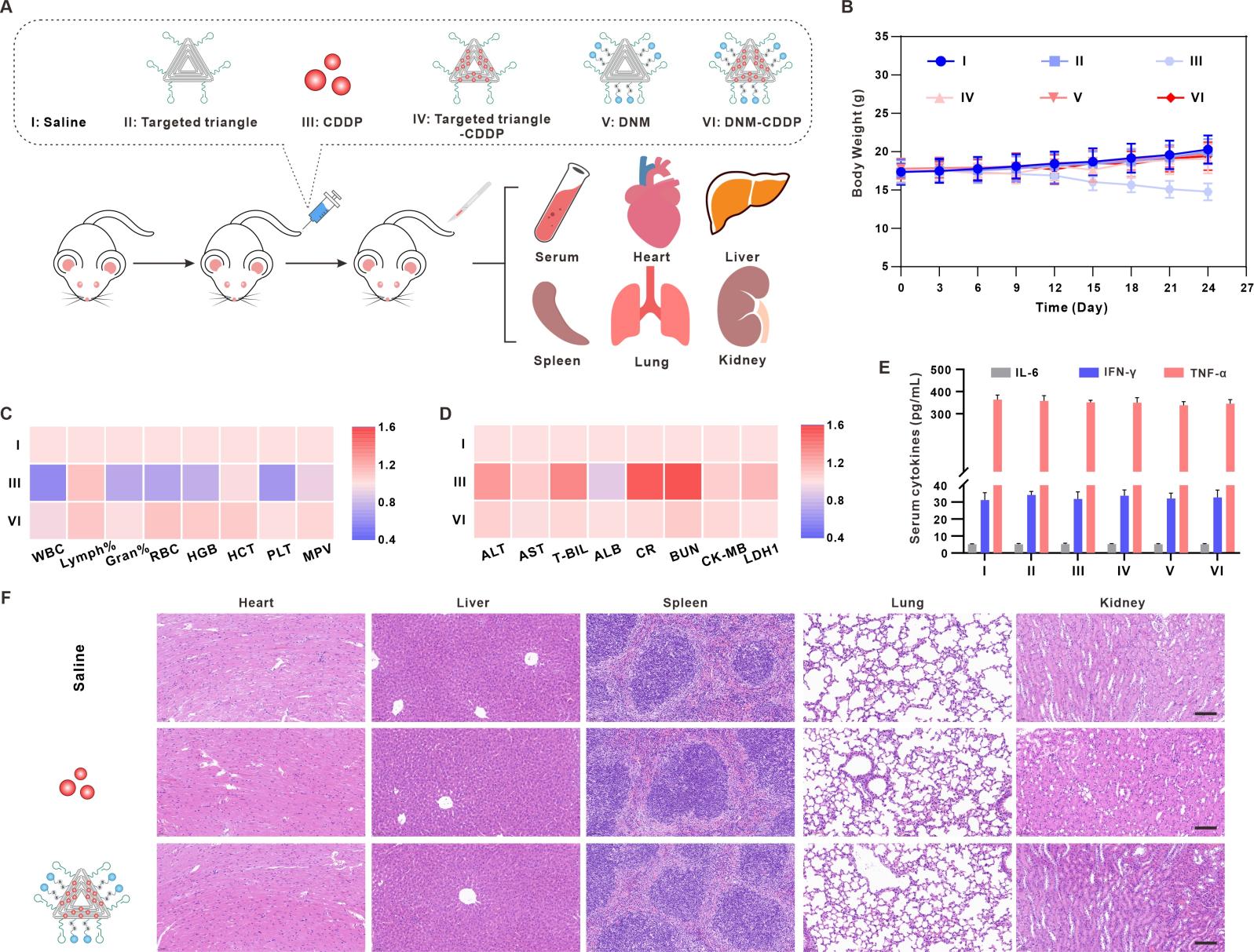
 Figure S15.** Biosafety investigation. (A) Schematic diagram of biosafety investigation process of different treatment groups. (B) Body weight change curves of mice after indicated treatments. (C, D) Heatmap of routine blood examinations (C) and blood biochemistry tests (D) in normal BALB/c mice treated with different groups. (E) Immune response of normal BALB/c mice treated with indicated groups by examining the cytokine levels including IL-6, IFN-γ, and TNF-α in the blood samples, respectively. (F) Representative histological images of key organs collected from normal BALB/c mice at 24-day post-initiation of different treatments. Scale bars, 100 µm. Data are shown as mean ± S.D. *n*= 3.

**Table S1.** DNA sequences of the staple strands of DNM.


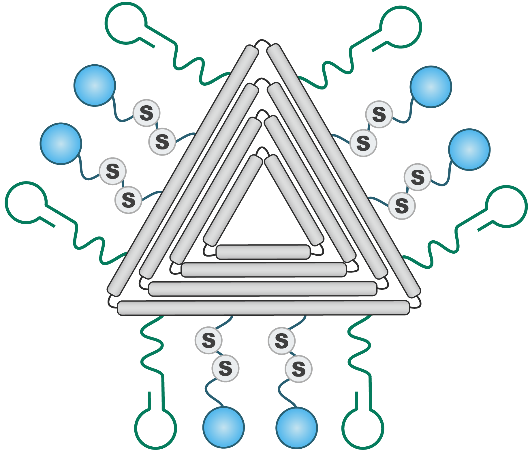


Name Sequences (5’ to 3’)

A01 CGGGGTTTCCTCAAGAGAAGGATTTTGAATTA

A02 AGCGTCATGTCTCTGAATTTACCGACTACCTT

A03 TTCATAATCCCCTTATTAGCGTTTTTCTTACC

A04 ATGGTTTATGTCACAATCAATAGATATTAAAC

A05 TTTGATGATTAAGAGGCTGAGACTTGCTCAGTACCAGGCG

A06 CCGGAACCCAGAATGGAAAGCGCAACATGGCT

A07 AAAGACAACATTTTCGGTCATAGCCAAAATCA

A08 GACGGGAGAATTAACTCGGAATAAGTTTATTTCCAGCGCC

A09 GATAAGTGCCGTCGAGCTGAAACATGAAAGTATACAGGAG

A10 TGTACTGGAAATCCTCATTAAAGCAGAGCCAC

A11 CACCGGAAAGCGCGTTTTCATCGGAAGGGCGA

A12 CATTCAACAAACGCAAAGACACCAGAACACCCTGAACAAA

A13 TTTAACGGTTCGGAACCTATTATTAGGGTTGATATAAGTA

A14 CTCAGAGCATATTCACAAACAAATTAATAAGT

A15 GGAGGGAATTTAGCGTCAGACTGTCCGCCTCC

A16-S-S-DCLX069

DCLX069-AATAA**S-S**GTCAGAGGGTAATTGATGGCAACATATAAAAGCGATTGAG

A17 TAGCCCGGAATAGGTGAATGCCCCCTGCCTATGGTCAGTG

A18 CCTTGAGTCAGACGATTGGCCTTGCGCCACCC

A19 TCAGAACCCAGAATCAAGTTTGCCGGTAAATA

A20 TTGACGGAAATACATACATAAAGGGCGCTAATATCAGAGA

A21 CAGAGCCAGGAGGTTGAGGCAGGTAACAGTGCCCG

A22 ATTAAAGGCCGTAATCAGTAGCGAGCCACCCT

A23 GATAACCCACAAGAATGTTAGCAAACGTAGAAAATTATTC

A24 GCCGCCAGCATTGACACCACCCTC

A25 AGAGCCGCACCATCGATAGCAGCATGAATTAT

A26 CACCGTCACCTTATTACGCAGTATTGAGTTAAGCCCAATA

A27 AGCCATTTAAACGTCACCAATGAACACCAGAACCA

A28 ATAAGAGCAAGAAACATGGCATGATTAAGACTCCGACTTG

A29 CCATTAGCAAGGCCGGGGGAATTA

A30 GAGCCAGCGAATACCCAAAAGAACATGAAATAGCAATAGC

A31-CD44 aptamer ACCGGGCGTACACCGTCGCGGCACATGTCTGAATGCGTTTAGTCTCTGTGAAAAATATCTTACCGAAGCCCAAACGCAATAATAACGAAAATCACCAG

A32 CAGAAGGAAACCGAGGTTTTTAAGAAAAGTAAGCAGATAGCCG

A33 CCTTTTTTCATTTAACAATTTCATAGGATTAG

A34 TTTAACCTATCATAGGTCTGAGAGTTCCAGTA

A35 AGTATAAAATATGCGTTATACAAAGCCATCTT

A36 CAAGTACCTCATTCCAAGAACGGGAAATTCAT

A37 AGAGAATAACATAAAAACAGGGAAGCGCATTA

A38 AAAACAAAATTAATTAAATGGAAACAGTACATTAGTGAAT

A39 TTATCAAACCGGCTTAGGTTGGGTAAGCCTGT

A40 TTAGTATCGCCAACGCTCAACAGTCGGCTGTC

A41 TTTCCTTAGCACTCATCGAGAACAATAGCAGCCTTTACAGS30

A42 AGAGTCAAAAATCAATATATGTGATGAAACAAACATCAAG

A43 ACTAGAAATATATAACTATATGTACGCTGAGA

A44 TCAATAATAGGGCTTAATTGAGAATCATAATT

A45-S-S-DCLX069

DCLX069-AATAA**S-S**AACGTCAAAAATGAAAAGCAAGCCGTTTTTATGAAACCAA

A46 GAGCAAAAGAAGATGAGTGAATAACCTTGCTTATAGCTTA

A47 GATTAAGAAATGCTGATGCAAATCAGAATAAA

A48 CACCGGAATCGCCATATTTAACAAAATTTACG

A49 AGCATGTATTTCATCGTAGGAATCAAACGATTTTTTGTTT

A50 ACATAGCGCTGTAAATCGTCGCTATTCATTTCAATTACCT

A51 GTTAAATACAATCGCAAGACAAAGCCTTGAAA

A52 CCCATCCTCGCCAACATGTAATTTAATAAGGC

A53 TCCCAATCCAAATAAGATTACCGCGCCCAATAAATAATAT

A54 TCCCTTAGAATAACGCGAGAAAACTTTTACCGACC

A55 GTGTGATAAGGCAGAGGCATTTTCAGTCCTGA

A56 ACAAGAAAGCAAGCAAATCAGATAACAGCCATATTATTTA

A57 GTTTGAAATTCAAATATATTTTAG

A58 AATAGATAGAGCCAGTAATAAGAGATTTAATG

A59 GCCAGTTACAAAATAATAGAAGGCTTATCCGGTTATCAAC

A60 TTCTGACCTAAAATATAAAGTACCGACTGCAGAAC

A61 GCGCCTGTTATTCTAAGAACGCGATTCCAGAGCCTAATTT

A62 TCAGCTAAAAAAGGTAAAGTAATT

A63-CD44 aptamer ACCGGGCGTACACCGTCGCGGCACATGTCTGAATGCGTTTAGTCTCTGTGAAAAAACGCTAACGAGCGTCTGGCGTTTTAGCGAACCCAACATGT

A64 ACGACAATAAATCCCGACTTGCGGGAGATCCTGAATCTTACCA

A65 TGCTATTTTGCACCCAGCTACAATTTTGTTTTGAAGCCTTAAA

B01 TCATATGTGTAATCGTAAAACTAGTCATTTTC

B02 GTGAGAAAATGTGTAGGTAAAGATACAACTTT

B03 GGCATCAAATTTGGGGCGCGAGCTAGTTAAAG

B04 TTCGAGCTAAGACTTCAAATATCGGGAACGAG

B05 ACAGTCAAAGAGAATCGATGAACGACCCCGGTTGATAATC

B06 ATAGTAGTATGCAATGCCTGAGTAGGCCGGAG

B07 AACCAGACGTTTAGCTATATTTTCTTCTACTA

B08 GAATACCACATTCAACTTAAGAGGAAGCCCGATCAAAGCG

B09 AGAAAAGCCCCAAAAAGAGTCTGGAGCAAACAATCACCAT

B10 CAATATGACCCTCATATATTTTAAAGCATTAA

B11 CATCCAATAAATGGTCAATAACCTCGGAAGCA

B12 AACTCCAAGATTGCATCAAAAAGATAATGCAGATACATAA

B13 CGTTCTAGTCAGGTCATTGCCTGACAGGAAGATTGTATAA

B14 CAGGCAAGATAAAAATTTTTAGAATATTCAAC

B15 GATTAGAGATTAGATACATTTCGCAAATCATA

B16-S-S-DCLX069

DCLX069-AATAA**S-S**CGCCAAAAGGAATTACAGTCAGAAGCAAAGCGCAGGTCAG

B17 GCAAATATTTAAATTGAGATCTACAAAGGCTACTGATAAA

B18 TTAATGCCTTATTTCAACGCAAGGGCAAAGAA

B19 TTAGCAAATAGATTTAGTTTGACCAGTACCTT

B20 TAATTGCTTTACCCTGACTATTATGAGGCATAGTAAGAGCS31

B21 ATAAAGCCTTTGCGGGAGAAGCCTGGAGAGGGTAG

B22 TAAGAGGTCAATTCTGCGAACGAGATTAAGCA

B23 AACACTATCATAACCCATCAAAAATCAGGTCTCCTTTTGA

B24 ATGACCCTGTAATACTTCAGAGCA

B25 TAAAGCTATATAACAGTTGATTCCCATTTTTG

B26 CGGATGGCACGAGAATGACCATAATCGTTTACCAGACGAC

B27 TAATTGCTTGGAAGTTTCATTCCAAATCGGTTGTA

B28 GATAAAAACCAAAATATTAAACAGTTCAGAAATTAGAGCT

B29 ACTAAAGTACGGTGTCGAATATAA

B30 TGCTGTAGATCCCCCTCAAATGCTGCGAGAGGCTTTTGCA

B31-CD44 aptamer ACCGGGCGTACACCGTCGCGGCACATGTCTGAATGCGTTTAGTCTCTGTGAAAAAAAAGAAGTTTTGCCAGCATAAATATTCATTGACTCAACATGTT

B32 AATACTGCGGAATCGTAGGGGGTAATAGTAAAATGTTTAGACT

B33 AGGGATAGCTCAGAGCCACCACCCCATGTCAA

B34 CAACAGTTTATGGGATTTTGCTAATCAAAAGG

B35 GCCGCTTTGCTGAGGCTTGCAGGGGAAAAGGT

B36 GCGCAGACTCCATGTTACTTAGCCCGTTTTAA

B37 ACAGGTAGAAAGATTCATCAGTTGAGATTTAG

B38 CCTCAGAACCGCCACCCAAGCCCAATAGGAACGTAAATGA

B39 ATTTTCTGTCAGCGGAGTGAGAATACCGATAT

B40 ATTCGGTCTGCGGGATCGTCACCCGAAATCCG

B41 CGACCTGCGGTCAATCATAAGGGAACGGAACAACATTATT

B42 AGACGTTACCATGTACCGTAACACCCCTCAGAACCGCCAC

B43 CACGCATAAGAAAGGAACAACTAAGTCTTTCC

B44 ATTGTGTCTCAGCAGCGAAAGACACCATCGCC

B45-S-S-DCLX069

DCLX069-AATAA**S-S**TTAATAAAACGAACTAACCGAACTGACCAACTCCTGATAA

B46 AGGTTTAGTACCGCCATGAGTTTCGTCACCAGGATCTAAA

B47 GTTTTGTCAGGAATTGCGAATAATCCGACAAT

B48 GACAACAAGCATCGGAACGAGGGTGAGATTTG

B49 TATCATCGTTGAAAGAGGACAGATGGAAGAAAAATCTACG

B50 AGCGTAACTACAAACTACAACGCCTATCACCGTACTCAGG

B51 TAGTTGCGAATTTTTTCACGTTGATCATAGTT

B52 GTACAACGAGCAACGGCTACAGAGGATACCGA

B53 ACCAGTCAGGACGTTGGAACGGTGTACAGACCGAAACAAA

B54 ACAGACAGCCCAAATCTCCAAAAAAAAATTTCTTA

B55 AACAGCTTGCTTTGAGGACTAAAGCGATTATA

B56 CCAAGCGCAGGCGCATAGGCTGGCAGAACTGGCTCATTAT

B57 CGAGGTGAGGCTCCAAAAGGAGCC

B58 ACCCCCAGACTTTTTCATGAGGAACTTGCTTT

B59 ACCTTATGCGATTTTATGACCTTCATCAAGAGCATCTTTG

B60 CGGTTTATCAGGTTTCCATTAAACGGGAATACACT

B61 AAAACACTTAATCTTGACAAGAACTTAATCATTGTGAATT

B62 GGCAAAAGTAAAATACGTAATGCC

B63-CD44 aptamer ACCGGGCGTACACCGTCGCGGCACATGTCTGAATGCGTTTAGTCTCTGTGAAAAAATGGTTTAATTTCAACTCGGATATTCATTACCCACGAAAGA

B64 ACCAACCTAAAAAATCAACGTAACAAATAAATTGGGCTTGAGAS32

B65 CCTGACGAGAAACACCAGAACGAGTAGGCTGCTCATTCAGTGA

Link-A1C TTAATTAATTTTTTACCATATCAAA

Link-A2C TTAATTTCATCTTAGACTTTACAA

Link-A3C CTGTCCAGACGTATACCGAACGA

Link-A4C TCAAGATTAGTGTAGCAATACT

Link-B1A TGTAGCATTCCTTTTATAAACAGTT

Link-B2A TTTAATTGTATTTCCACCAGAGCC

Link-B3A ACTACGAAGGCTTAGCACCATTA

Link-B4A ATAAGGCTTGCAACAAAGTTAC

Link-C1B GTGGGAACAAATTTCTATTTTTGAG

Link-C2B CGGTGCGGGCCTTCCAAAAACATT

Link-C3B ATGAGTGAGCTTTTAAATATGCA

Link-C4B ACTATTAAAGAGGATAGCGTCC

Loop GCGCTTAATGCGCCGCTACAGGGC

C01 TCGGGAGATATACAGTAACAGTACAAATAATT

C02 CCTGATTAAAGGAGCGGAATTATCTCGGCCTC

C03 GCAAATCACCTCAATCAATATCTGCAGGTCGA

C04 CGACCAGTACATTGGCAGATTCACCTGATTGC

C05 TGGCAATTTTTAACGTCAGATGAAAACAATAACGGATTCG

C06 AAGGAATTACAAAGAAACCACCAGTCAGATGA

C07 GGACATTCACCTCAAATATCAAACACAGTTGA

C08 TTGACGAGCACGTATACTGAAATGGATTATTTAATAAAAG

C09 CCTGATTGCTTTGAATTGCGTAGATTTTCAGGCATCAATA

C10 TAATCCTGATTATCATTTTGCGGAGAGGAAGG

C11 TTATCTAAAGCATCACCTTGCTGATGGCCAAC

C12 AGAGATAGTTTGACGCTCAATCGTACGTGCTTTCCTCGTT

C13 GATTATACACAGAAATAAAGAAATACCAAGTTACAAAATC

C14 TAGGAGCATAAAAGTTTGAGTAACATTGTTTG

C15 TGACCTGACAAATGAAAAATCTAAAATATCTT

C16-S-S-DCLX069

DCLX069-AATAA**S-S**AGAATCAGAGCGGGAGATGGAAATACCTACATAACCCTTC

C17 GCGCAGAGGCGAATTAATTATTTGCACGTAAATTCTGAAT

C18 AATGGAAGCGAACGTTATTAATTTCTAACAAC

C19 TAATAGATCGCTGAGAGCCAGCAGAAGCGTAA

C20 GAATACGTAACAGGAAAAACGCTCCTAAACAGGAGGCCGA

C21 TCAATAGATATTAAATCCTTTGCCGGTTAGAACCT

C22 CAATATTTGCCTGCAACAGTGCCATAGAGCCG

C23 TTAAAGGGATTTTAGATACCGCCAGCCATTGCGGCACAGA

C24 ACAATTCGACAACTCGTAATACAT

C25 TTGAGGATGGTCAGTATTAACACCTTGAATGG

C26 CTATTAGTATATCCAGAACAATATCAGGAACGGTACGCCA

C27 CGCGAACTAAAACAGAGGTGAGGCTTAGAAGTATT

C28 GAATCCTGAGAAGTGTATCGGCCTTGCTGGTACTTTAATG

C29 ACCACCAGCAGAAGATGATAGCCC

C30 TAAAACATTAGAAGAACTCAAACTTTTTATAATCAGTGAGS33

C31-CD44 aptamer ACCGGGCGTACACCGTCGCGGCACATGTCTGAATGCGTTTAGTCTCTGTGAAAAAGCCACCGAGTAAAAGAACATCACTTGCCTGAGCGCCATTAAAA

C32 TCTTTGATTAGTAATAGTCTGTCCATCACGCAAATTAACCGTT

C33 CGCGTCTGATAGGAACGCCATCAACTTTTACA

C34 AGGAAGATGGGGACGACGACAGTAATCATATT

C35 CTCTAGAGCAAGCTTGCATGCCTGGTCAGTTG

C36 CCTTCACCGTGAGACGGGCAACAGCAGTCACA

C37 CGAGAAAGGAAGGGAAGCGTACTATGGTTGCT

C38 GCTCATTTTTTAACCAGCCTTCCTGTAGCCAGGCATCTGC

C39 CAGTTTGACGCACTCCAGCCAGCTAAACGACG

C40 GCCAGTGCGATCCCCGGGTACCGAGTTTTTCT

C41 TTTCACCAGCCTGGCCCTGAGAGAAAGCCGGCGAACGTGG

C42 GTAACCGTCTTTCATCAACATTAAAATTTTTGTTAAATCA

C43 ACGTTGTATTCCGGCACCGCTTCTGGCGCATC

C44 CCAGGGTGGCTCGAATTCGTAATCCAGTCACG

C45-S-S-DCLX069

DCLX069-AATAA**S-S**TAGAGCTTGACGGGGAGTTGCAGCAAGCGGTCATTGGGCG

C46 GTTAAAATTCGCATTAATGTGAGCGAGTAACACACGTTGG

C47 TGTAGATGGGTGCCGGAAACCAGGAACGCCAG

C48 GGTTTTCCATGGTCATAGCTGTTTGAGAGGCG

C49 GTTTGCGTCACGCTGGTTTGCCCCAAGGGAGCCCCCGATT

C50 GGATAGGTACCCGTCGGATTCTCCTAAACGTTAATATTTT

C51 AGTTGGGTCAAAGCGCCATTCGCCCCGTAATG

C52 CGCGCGGGCCTGTGTGAAATTGTTGGCGATTA

C53 CTAAATCGGAACCCTAAGCAGGCGAAAATCCTTCGGCCAA

C54 CGGCGGATTGAATTCAGGCTGCGCAACGGGGGATG

C55 TGCTGCAAATCCGCTCACAATTCCCAGCTGCA

C56 TTAATGAAGTTTGATGGTGGTTCCGAGGTGCCGTAAAGCA

C57 TGGCGAAATGTTGGGAAGGGCGAT

C58 TGTCGTGCACACAACATACGAGCCACGCCAGC

C59 CAAGTTTTTTGGGGTCGAAATCGGCAAAATCCGGGAAACC

C60 TCTTCGCTATTGGAAGCATAAAGTGTATGCCCGCT

C61 TTCCAGTCCTTATAAATCAAAAGAGAACCATCACCCAAAT

C62 GCGCTCACAAGCCTGGGGTGCCTA

C63-CD44 aptamer ACCGGGCGTACACCGTCGCGGCACATGTCTGAATGCGTTTAGTCTCTGTGAAAAACGATGGCCCACTACGTATAGCCCGAGATAGGGATTGCGTT

C64 AACTCACATTATTGAGTGTTGTTCCAGAAACCGTCTATCAGGG

C65 ACGTGGACTCCAACGTCAAAGGGCGAATTTGGAACAAGAGTCC

**Table S2.** DNA sequences of the staple strands of non-targeted DNM.


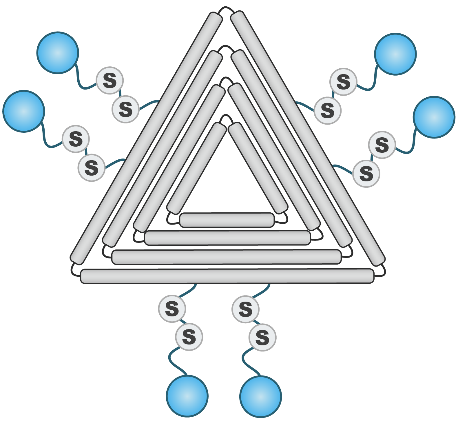


Name Sequences (5’ to 3’)

A01 CGGGGTTTCCTCAAGAGAAGGATTTTGAATTA

A02 AGCGTCATGTCTCTGAATTTACCGACTACCTT

A03 TTCATAATCCCCTTATTAGCGTTTTTCTTACC

A04 ATGGTTTATGTCACAATCAATAGATATTAAAC

A05 TTTGATGATTAAGAGGCTGAGACTTGCTCAGTACCAGGCG

A06 CCGGAACCCAGAATGGAAAGCGCAACATGGCT

A07 AAAGACAACATTTTCGGTCATAGCCAAAATCA

A08 GACGGGAGAATTAACTCGGAATAAGTTTATTTCCAGCGCC

A09 GATAAGTGCCGTCGAGCTGAAACATGAAAGTATACAGGAG

A10 TGTACTGGAAATCCTCATTAAAGCAGAGCCAC

A11 CACCGGAAAGCGCGTTTTCATCGGAAGGGCGA

A12 CATTCAACAAACGCAAAGACACCAGAACACCCTGAACAAA

A13 TTTAACGGTTCGGAACCTATTATTAGGGTTGATATAAGTA

A14 CTCAGAGCATATTCACAAACAAATTAATAAGT

A15 GGAGGGAATTTAGCGTCAGACTGTCCGCCTCC

A16-S-S-DCLX069

DCLX069-AATAA**S-S**GTCAGAGGGTAATTGATGGCAACATATAAAAGCGATTGAG

A17 TAGCCCGGAATAGGTGAATGCCCCCTGCCTATGGTCAGTG

A18 CCTTGAGTCAGACGATTGGCCTTGCGCCACCC

A19 TCAGAACCCAGAATCAAGTTTGCCGGTAAATA

A20 TTGACGGAAATACATACATAAAGGGCGCTAATATCAGAGA

A21 CAGAGCCAGGAGGTTGAGGCAGGTAACAGTGCCCG

A22 ATTAAAGGCCGTAATCAGTAGCGAGCCACCCT

A23 GATAACCCACAAGAATGTTAGCAAACGTAGAAAATTATTC

A24 GCCGCCAGCATTGACACCACCCTC

A25 AGAGCCGCACCATCGATAGCAGCATGAATTAT

A26 CACCGTCACCTTATTACGCAGTATTGAGTTAAGCCCAATA

A27 AGCCATTTAAACGTCACCAATGAACACCAGAACCA

A28 ATAAGAGCAAGAAACATGGCATGATTAAGACTCCGACTTG

A29 CCATTAGCAAGGCCGGGGGAATTA

A30 GAGCCAGCGAATACCCAAAAGAACATGAAATAGCAATAGC

A31 TATCTTACCGAAGCCCAAACGCAATAATAACGAAAATCACCAG

A32 CAGAAGGAAACCGAGGTTTTTAAGAAAAGTAAGCAGATAGCCG

A33 CCTTTTTTCATTTAACAATTTCATAGGATTAG

A34 TTTAACCTATCATAGGTCTGAGAGTTCCAGTA

A35 AGTATAAAATATGCGTTATACAAAGCCATCTT

A36 CAAGTACCTCATTCCAAGAACGGGAAATTCAT

A37 AGAGAATAACATAAAAACAGGGAAGCGCATTA

A38 AAAACAAAATTAATTAAATGGAAACAGTACATTAGTGAAT

A39 TTATCAAACCGGCTTAGGTTGGGTAAGCCTGT

A40 TTAGTATCGCCAACGCTCAACAGTCGGCTGTC

A41 TTTCCTTAGCACTCATCGAGAACAATAGCAGCCTTTACAGS30

A42 AGAGTCAAAAATCAATATATGTGATGAAACAAACATCAAG

A43 ACTAGAAATATATAACTATATGTACGCTGAGA

A44 TCAATAATAGGGCTTAATTGAGAATCATAATT

A45-S-S-DCLX069

DCLX069-AATAA**S-S**AACGTCAAAAATGAAAAGCAAGCCGTTTTTATGAAACCAA

A46 GAGCAAAAGAAGATGAGTGAATAACCTTGCTTATAGCTTA

A47 GATTAAGAAATGCTGATGCAAATCAGAATAAA

A48 CACCGGAATCGCCATATTTAACAAAATTTACG

A49 AGCATGTATTTCATCGTAGGAATCAAACGATTTTTTGTTT

A50 ACATAGCGCTGTAAATCGTCGCTATTCATTTCAATTACCT

A51 GTTAAATACAATCGCAAGACAAAGCCTTGAAA

A52 CCCATCCTCGCCAACATGTAATTTAATAAGGC

A53 TCCCAATCCAAATAAGATTACCGCGCCCAATAAATAATAT

A54 TCCCTTAGAATAACGCGAGAAAACTTTTACCGACC

A55 GTGTGATAAGGCAGAGGCATTTTCAGTCCTGA

A56 ACAAGAAAGCAAGCAAATCAGATAACAGCCATATTATTTA

A57 GTTTGAAATTCAAATATATTTTAG

A58 AATAGATAGAGCCAGTAATAAGAGATTTAATG

A59 GCCAGTTACAAAATAATAGAAGGCTTATCCGGTTATCAAC

A60 TTCTGACCTAAAATATAAAGTACCGACTGCAGAAC

A61 GCGCCTGTTATTCTAAGAACGCGATTCCAGAGCCTAATTT

A62 TCAGCTAAAAAAGGTAAAGTAATT

A63 ACGCTAACGAGCGTCTGGCGTTTTAGCGAACCCAACATGT

A64 ACGACAATAAATCCCGACTTGCGGGAGATCCTGAATCTTACCA

A65 TGCTATTTTGCACCCAGCTACAATTTTGTTTTGAAGCCTTAAA

B01 TCATATGTGTAATCGTAAAACTAGTCATTTTC

B02 GTGAGAAAATGTGTAGGTAAAGATACAACTTT

B03 GGCATCAAATTTGGGGCGCGAGCTAGTTAAAG

B04 TTCGAGCTAAGACTTCAAATATCGGGAACGAG

B05 ACAGTCAAAGAGAATCGATGAACGACCCCGGTTGATAATC

B06 ATAGTAGTATGCAATGCCTGAGTAGGCCGGAG

B07 AACCAGACGTTTAGCTATATTTTCTTCTACTA

B08 GAATACCACATTCAACTTAAGAGGAAGCCCGATCAAAGCG

B09 AGAAAAGCCCCAAAAAGAGTCTGGAGCAAACAATCACCAT

B10 CAATATGACCCTCATATATTTTAAAGCATTAA

B11 CATCCAATAAATGGTCAATAACCTCGGAAGCA

B12 AACTCCAAGATTGCATCAAAAAGATAATGCAGATACATAA

B13 CGTTCTAGTCAGGTCATTGCCTGACAGGAAGATTGTATAA

B14 CAGGCAAGATAAAAATTTTTAGAATATTCAAC

B15 GATTAGAGATTAGATACATTTCGCAAATCATA

B16-S-S-DCLX069

DCLX069-AATAA**S-S**CGCCAAAAGGAATTACAGTCAGAAGCAAAGCGCAGGTCAG

B17 GCAAATATTTAAATTGAGATCTACAAAGGCTACTGATAAA

B18 TTAATGCCTTATTTCAACGCAAGGGCAAAGAA

B19 TTAGCAAATAGATTTAGTTTGACCAGTACCTT

B20 TAATTGCTTTACCCTGACTATTATGAGGCATAGTAAGAGCS31

B21 ATAAAGCCTTTGCGGGAGAAGCCTGGAGAGGGTAG

B22 TAAGAGGTCAATTCTGCGAACGAGATTAAGCA

B23 AACACTATCATAACCCATCAAAAATCAGGTCTCCTTTTGA

B24 ATGACCCTGTAATACTTCAGAGCA

B25 TAAAGCTATATAACAGTTGATTCCCATTTTTG

B26 CGGATGGCACGAGAATGACCATAATCGTTTACCAGACGAC

B27 TAATTGCTTGGAAGTTTCATTCCAAATCGGTTGTA

B28 GATAAAAACCAAAATATTAAACAGTTCAGAAATTAGAGCT

B29 ACTAAAGTACGGTGTCGAATATAA

B30 TGCTGTAGATCCCCCTCAAATGCTGCGAGAGGCTTTTGCA

B31 AAAGAAGTTTTGCCAGCATAAATATTCATTGACTCAACATGTT

B32 AATACTGCGGAATCGTAGGGGGTAATAGTAAAATGTTTAGACT

B33 AGGGATAGCTCAGAGCCACCACCCCATGTCAA

B34 CAACAGTTTATGGGATTTTGCTAATCAAAAGG

B35 GCCGCTTTGCTGAGGCTTGCAGGGGAAAAGGT

B36 GCGCAGACTCCATGTTACTTAGCCCGTTTTAA

B37 ACAGGTAGAAAGATTCATCAGTTGAGATTTAG

B38 CCTCAGAACCGCCACCCAAGCCCAATAGGAACGTAAATGA

B39 ATTTTCTGTCAGCGGAGTGAGAATACCGATAT

B40 ATTCGGTCTGCGGGATCGTCACCCGAAATCCG

B41 CGACCTGCGGTCAATCATAAGGGAACGGAACAACATTATT

B42 AGACGTTACCATGTACCGTAACACCCCTCAGAACCGCCAC

B43 CACGCATAAGAAAGGAACAACTAAGTCTTTCC

B44 ATTGTGTCTCAGCAGCGAAAGACACCATCGCC

B45-S-S-DCLX069

DCLX069-AATAA**S-S**TTAATAAAACGAACTAACCGAACTGACCAACTCCTGATAA

B46 AGGTTTAGTACCGCCATGAGTTTCGTCACCAGGATCTAAA

B47 GTTTTGTCAGGAATTGCGAATAATCCGACAAT

B48 GACAACAAGCATCGGAACGAGGGTGAGATTTG

B49 TATCATCGTTGAAAGAGGACAGATGGAAGAAAAATCTACG

B50 AGCGTAACTACAAACTACAACGCCTATCACCGTACTCAGG

B51 TAGTTGCGAATTTTTTCACGTTGATCATAGTT

B52 GTACAACGAGCAACGGCTACAGAGGATACCGA

B53 ACCAGTCAGGACGTTGGAACGGTGTACAGACCGAAACAAA

B54 ACAGACAGCCCAAATCTCCAAAAAAAAATTTCTTA

B55 AACAGCTTGCTTTGAGGACTAAAGCGATTATA

B56 CCAAGCGCAGGCGCATAGGCTGGCAGAACTGGCTCATTAT

B57 CGAGGTGAGGCTCCAAAAGGAGCC

B58 ACCCCCAGACTTTTTCATGAGGAACTTGCTTT

B59 ACCTTATGCGATTTTATGACCTTCATCAAGAGCATCTTTG

B60 CGGTTTATCAGGTTTCCATTAAACGGGAATACACT

B61 AAAACACTTAATCTTGACAAGAACTTAATCATTGTGAATT

B62 GGCAAAAGTAAAATACGTAATGCC

B63 TGGTTTAATTTCAACTCGGATATTCATTACCCACGAAAGA

B64 ACCAACCTAAAAAATCAACGTAACAAATAAATTGGGCTTGAGAS32

B65 CCTGACGAGAAACACCAGAACGAGTAGGCTGCTCATTCAGTGA

Link-A1C TTAATTAATTTTTTACCATATCAAA

Link-A2C TTAATTTCATCTTAGACTTTACAA

Link-A3C CTGTCCAGACGTATACCGAACGA

Link-A4C TCAAGATTAGTGTAGCAATACT

Link-B1A TGTAGCATTCCTTTTATAAACAGTT

Link-B2A TTTAATTGTATTTCCACCAGAGCC

Link-B3A ACTACGAAGGCTTAGCACCATTA

Link-B4A ATAAGGCTTGCAACAAAGTTAC

Link-C1B GTGGGAACAAATTTCTATTTTTGAG

Link-C2B CGGTGCGGGCCTTCCAAAAACATT

Link-C3B ATGAGTGAGCTTTTAAATATGCA

Link-C4B ACTATTAAAGAGGATAGCGTCC

Loop GCGCTTAATGCGCCGCTACAGGGC

C01 TCGGGAGATATACAGTAACAGTACAAATAATT

C02 CCTGATTAAAGGAGCGGAATTATCTCGGCCTC

C03 GCAAATCACCTCAATCAATATCTGCAGGTCGA

C04 CGACCAGTACATTGGCAGATTCACCTGATTGC

C05 TGGCAATTTTTAACGTCAGATGAAAACAATAACGGATTCG

C06 AAGGAATTACAAAGAAACCACCAGTCAGATGA

C07 GGACATTCACCTCAAATATCAAACACAGTTGA

C08 TTGACGAGCACGTATACTGAAATGGATTATTTAATAAAAG

C09 CCTGATTGCTTTGAATTGCGTAGATTTTCAGGCATCAATA

C10 TAATCCTGATTATCATTTTGCGGAGAGGAAGG

C11 TTATCTAAAGCATCACCTTGCTGATGGCCAAC

C12 AGAGATAGTTTGACGCTCAATCGTACGTGCTTTCCTCGTT

C13 GATTATACACAGAAATAAAGAAATACCAAGTTACAAAATC

C14 TAGGAGCATAAAAGTTTGAGTAACATTGTTTG

C15 TGACCTGACAAATGAAAAATCTAAAATATCTT

C16-S-S-DCLX069

DCLX069-AATAA**S-S**AGAATCAGAGCGGGAGATGGAAATACCTACATAACCCTTC

C17 GCGCAGAGGCGAATTAATTATTTGCACGTAAATTCTGAAT

C18 AATGGAAGCGAACGTTATTAATTTCTAACAAC

C19 TAATAGATCGCTGAGAGCCAGCAGAAGCGTAA

C20 GAATACGTAACAGGAAAAACGCTCCTAAACAGGAGGCCGA

C21 TCAATAGATATTAAATCCTTTGCCGGTTAGAACCT

C22 CAATATTTGCCTGCAACAGTGCCATAGAGCCG

C23 TTAAAGGGATTTTAGATACCGCCAGCCATTGCGGCACAGA

C24 ACAATTCGACAACTCGTAATACAT

C25 TTGAGGATGGTCAGTATTAACACCTTGAATGG

C26 CTATTAGTATATCCAGAACAATATCAGGAACGGTACGCCA

C27 CGCGAACTAAAACAGAGGTGAGGCTTAGAAGTATT

C28 GAATCCTGAGAAGTGTATCGGCCTTGCTGGTACTTTAATG

C29 ACCACCAGCAGAAGATGATAGCCC

C30 TAAAACATTAGAAGAACTCAAACTTTTTATAATCAGTGAGS33

C31 GCCACCGAGTAAAAGAACATCACTTGCCTGAGCGCCATTAAAA

C32 TCTTTGATTAGTAATAGTCTGTCCATCACGCAAATTAACCGTT

C33 CGCGTCTGATAGGAACGCCATCAACTTTTACA

C34 AGGAAGATGGGGACGACGACAGTAATCATATT

C35 CTCTAGAGCAAGCTTGCATGCCTGGTCAGTTG

C36 CCTTCACCGTGAGACGGGCAACAGCAGTCACA

C37 CGAGAAAGGAAGGGAAGCGTACTATGGTTGCT

C38 GCTCATTTTTTAACCAGCCTTCCTGTAGCCAGGCATCTGC

C39 CAGTTTGACGCACTCCAGCCAGCTAAACGACG

C40 GCCAGTGCGATCCCCGGGTACCGAGTTTTTCT

C41 TTTCACCAGCCTGGCCCTGAGAGAAAGCCGGCGAACGTGG

C42 GTAACCGTCTTTCATCAACATTAAAATTTTTGTTAAATCA

C43 ACGTTGTATTCCGGCACCGCTTCTGGCGCATC

C44 CCAGGGTGGCTCGAATTCGTAATCCAGTCACG

C45-S-S-DCLX069

DCLX069-AATAA**S-S**TAGAGCTTGACGGGGAGTTGCAGCAAGCGGTCATTGGGCG

C46 GTTAAAATTCGCATTAATGTGAGCGAGTAACACACGTTGG

C47 TGTAGATGGGTGCCGGAAACCAGGAACGCCAG

C48 GGTTTTCCATGGTCATAGCTGTTTGAGAGGCG

C49 GTTTGCGTCACGCTGGTTTGCCCCAAGGGAGCCCCCGATT

C50 GGATAGGTACCCGTCGGATTCTCCTAAACGTTAATATTTT

C51 AGTTGGGTCAAAGCGCCATTCGCCCCGTAATG

C52 CGCGCGGGCCTGTGTGAAATTGTTGGCGATTA

C53 CTAAATCGGAACCCTAAGCAGGCGAAAATCCTTCGGCCAA

C54 CGGCGGATTGAATTCAGGCTGCGCAACGGGGGATG

C55 TGCTGCAAATCCGCTCACAATTCCCAGCTGCA

C56 TTAATGAAGTTTGATGGTGGTTCCGAGGTGCCGTAAAGCA

C57 TGGCGAAATGTTGGGAAGGGCGAT

C58 TGTCGTGCACACAACATACGAGCCACGCCAGC

C59 CAAGTTTTTTGGGGTCGAAATCGGCAAAATCCGGGAAACC

C60 TCTTCGCTATTGGAAGCATAAAGTGTATGCCCGCT

C61 TTCCAGTCCTTATAAATCAAAAGAGAACCATCACCCAAAT

C62 GCGCTCACAAGCCTGGGGTGCCTA

C63 CGATGGCCCACTACGTATAGCCCGAGATAGGGATTGCGTT

C64 AACTCACATTATTGAGTGTTGTTCCAGAAACCGTCTATCAGGG

C65 ACGTGGACTCCAACGTCAAAGGGCGAATTTGGAACAAGAGTCC

**Table S3.** DNA sequences of the staple strands of the targeted triangle.


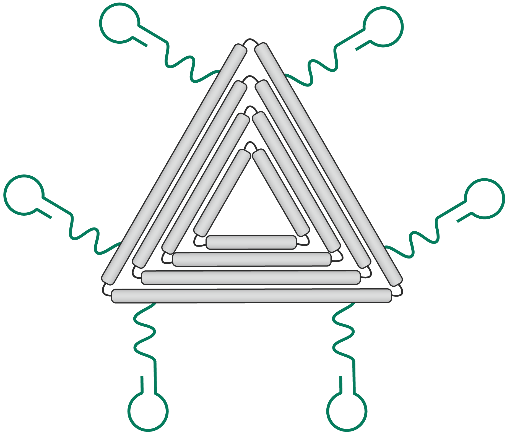


Name Sequences (5’ to 3’)

A01 CGGGGTTTCCTCAAGAGAAGGATTTTGAATTA

A02 AGCGTCATGTCTCTGAATTTACCGACTACCTT

A03 TTCATAATCCCCTTATTAGCGTTTTTCTTACC

A04 ATGGTTTATGTCACAATCAATAGATATTAAAC

A05 TTTGATGATTAAGAGGCTGAGACTTGCTCAGTACCAGGCG

A06 CCGGAACCCAGAATGGAAAGCGCAACATGGCT

A07 AAAGACAACATTTTCGGTCATAGCCAAAATCA

A08 GACGGGAGAATTAACTCGGAATAAGTTTATTTCCAGCGCC

A09 GATAAGTGCCGTCGAGCTGAAACATGAAAGTATACAGGAG

A10 TGTACTGGAAATCCTCATTAAAGCAGAGCCAC

A11 CACCGGAAAGCGCGTTTTCATCGGAAGGGCGA

A12 CATTCAACAAACGCAAAGACACCAGAACACCCTGAACAAA

A13 TTTAACGGTTCGGAACCTATTATTAGGGTTGATATAAGTA

A14 CTCAGAGCATATTCACAAACAAATTAATAAGT

A15 GGAGGGAATTTAGCGTCAGACTGTCCGCCTCC

A16 GTCAGAGGGTAATTGATGGCAACATATAAAAGCGATTGAG

A17 TAGCCCGGAATAGGTGAATGCCCCCTGCCTATGGTCAGTG

A18 CCTTGAGTCAGACGATTGGCCTTGCGCCACCC

A19 TCAGAACCCAGAATCAAGTTTGCCGGTAAATA

A20 TTGACGGAAATACATACATAAAGGGCGCTAATATCAGAGA

A21 CAGAGCCAGGAGGTTGAGGCAGGTAACAGTGCCCG

A22 ATTAAAGGCCGTAATCAGTAGCGAGCCACCCT

A23 GATAACCCACAAGAATGTTAGCAAACGTAGAAAATTATTC

A24 GCCGCCAGCATTGACACCACCCTC

A25 AGAGCCGCACCATCGATAGCAGCATGAATTAT

A26 CACCGTCACCTTATTACGCAGTATTGAGTTAAGCCCAATA

A27 AGCCATTTAAACGTCACCAATGAACACCAGAACCA

A28 ATAAGAGCAAGAAACATGGCATGATTAAGACTCCGACTTG

A29 CCATTAGCAAGGCCGGGGGAATTA

A30 GAGCCAGCGAATACCCAAAAGAACATGAAATAGCAATAGC

A31-CD44 aptamer ACCGGGCGTACACCGTCGCGGCACATGTCTGAATGCGTTTAGTCTCTGTGAAAAATATCTTACCGAAGCCCAAACGCAATAATAACGAAAATCACCAG

A32 CAGAAGGAAACCGAGGTTTTTAAGAAAAGTAAGCAGATAGCCG

A33 CCTTTTTTCATTTAACAATTTCATAGGATTAG

A34 TTTAACCTATCATAGGTCTGAGAGTTCCAGTA

A35 AGTATAAAATATGCGTTATACAAAGCCATCTT

A36 CAAGTACCTCATTCCAAGAACGGGAAATTCAT

A37 AGAGAATAACATAAAAACAGGGAAGCGCATTA

A38 AAAACAAAATTAATTAAATGGAAACAGTACATTAGTGAAT

A39 TTATCAAACCGGCTTAGGTTGGGTAAGCCTGT

A40 TTAGTATCGCCAACGCTCAACAGTCGGCTGTC

A41 TTTCCTTAGCACTCATCGAGAACAATAGCAGCCTTTACAGS30

A42 AGAGTCAAAAATCAATATATGTGATGAAACAAACATCAAG

A43 ACTAGAAATATATAACTATATGTACGCTGAGA

A44 TCAATAATAGGGCTTAATTGAGAATCATAATT

A45 AACGTCAAAAATGAAAAGCAAGCCGTTTTTATGAAACCAA

A46 GAGCAAAAGAAGATGAGTGAATAACCTTGCTTATAGCTTA

A47 GATTAAGAAATGCTGATGCAAATCAGAATAAA

A48 CACCGGAATCGCCATATTTAACAAAATTTACG

A49 AGCATGTATTTCATCGTAGGAATCAAACGATTTTTTGTTT

A50 ACATAGCGCTGTAAATCGTCGCTATTCATTTCAATTACCT

A51 GTTAAATACAATCGCAAGACAAAGCCTTGAAA

A52 CCCATCCTCGCCAACATGTAATTTAATAAGGC

A53 TCCCAATCCAAATAAGATTACCGCGCCCAATAAATAATAT

A54 TCCCTTAGAATAACGCGAGAAAACTTTTACCGACC

A55 GTGTGATAAGGCAGAGGCATTTTCAGTCCTGA

A56 ACAAGAAAGCAAGCAAATCAGATAACAGCCATATTATTTA

A57 GTTTGAAATTCAAATATATTTTAG

A58 AATAGATAGAGCCAGTAATAAGAGATTTAATG

A59 GCCAGTTACAAAATAATAGAAGGCTTATCCGGTTATCAAC

A60 TTCTGACCTAAAATATAAAGTACCGACTGCAGAAC

A61 GCGCCTGTTATTCTAAGAACGCGATTCCAGAGCCTAATTT

A62 TCAGCTAAAAAAGGTAAAGTAATT

A63-CD44 aptamer ACCGGGCGTACACCGTCGCGGCACATGTCTGAATGCGTTTAGTCTCTGTGAAAAAACGCTAACGAGCGTCTGGCGTTTTAGCGAACCCAACATGT

A64 ACGACAATAAATCCCGACTTGCGGGAGATCCTGAATCTTACCA

A65 TGCTATTTTGCACCCAGCTACAATTTTGTTTTGAAGCCTTAAA

B01 TCATATGTGTAATCGTAAAACTAGTCATTTTC

B02 GTGAGAAAATGTGTAGGTAAAGATACAACTTT

B03 GGCATCAAATTTGGGGCGCGAGCTAGTTAAAG

B04 TTCGAGCTAAGACTTCAAATATCGGGAACGAG

B05 ACAGTCAAAGAGAATCGATGAACGACCCCGGTTGATAATC

B06 ATAGTAGTATGCAATGCCTGAGTAGGCCGGAG

B07 AACCAGACGTTTAGCTATATTTTCTTCTACTA

B08 GAATACCACATTCAACTTAAGAGGAAGCCCGATCAAAGCG

B09 AGAAAAGCCCCAAAAAGAGTCTGGAGCAAACAATCACCAT

B10 CAATATGACCCTCATATATTTTAAAGCATTAA

B11 CATCCAATAAATGGTCAATAACCTCGGAAGCA

B12 AACTCCAAGATTGCATCAAAAAGATAATGCAGATACATAA

B13 CGTTCTAGTCAGGTCATTGCCTGACAGGAAGATTGTATAA

B14 CAGGCAAGATAAAAATTTTTAGAATATTCAAC

B15 GATTAGAGATTAGATACATTTCGCAAATCATA

B16 CGCCAAAAGGAATTACAGTCAGAAGCAAAGCGCAGGTCAG

B17 GCAAATATTTAAATTGAGATCTACAAAGGCTACTGATAAA

B18 TTAATGCCTTATTTCAACGCAAGGGCAAAGAA

B19 TTAGCAAATAGATTTAGTTTGACCAGTACCTT

B20 TAATTGCTTTACCCTGACTATTATGAGGCATAGTAAGAGCS31

B21 ATAAAGCCTTTGCGGGAGAAGCCTGGAGAGGGTAG

B22 TAAGAGGTCAATTCTGCGAACGAGATTAAGCA

B23 AACACTATCATAACCCATCAAAAATCAGGTCTCCTTTTGA

B24 ATGACCCTGTAATACTTCAGAGCA

B25 TAAAGCTATATAACAGTTGATTCCCATTTTTG

B26 CGGATGGCACGAGAATGACCATAATCGTTTACCAGACGAC

B27 TAATTGCTTGGAAGTTTCATTCCAAATCGGTTGTA

B28 GATAAAAACCAAAATATTAAACAGTTCAGAAATTAGAGCT

B29 ACTAAAGTACGGTGTCGAATATAA

B30 TGCTGTAGATCCCCCTCAAATGCTGCGAGAGGCTTTTGCA

B31-CD44 aptamer ACCGGGCGTACACCGTCGCGGCACATGTCTGAATGCGTTTAGTCTCTGTGAAAAAAAAGAAGTTTTGCCAGCATAAATATTCATTGACTCAACATGTT

B32 AATACTGCGGAATCGTAGGGGGTAATAGTAAAATGTTTAGACT

B33 AGGGATAGCTCAGAGCCACCACCCCATGTCAA

B34 CAACAGTTTATGGGATTTTGCTAATCAAAAGG

B35 GCCGCTTTGCTGAGGCTTGCAGGGGAAAAGGT

B36 GCGCAGACTCCATGTTACTTAGCCCGTTTTAA

B37 ACAGGTAGAAAGATTCATCAGTTGAGATTTAG

B38 CCTCAGAACCGCCACCCAAGCCCAATAGGAACGTAAATGA

B39 ATTTTCTGTCAGCGGAGTGAGAATACCGATAT

B40 ATTCGGTCTGCGGGATCGTCACCCGAAATCCG

B41 CGACCTGCGGTCAATCATAAGGGAACGGAACAACATTATT

B42 AGACGTTACCATGTACCGTAACACCCCTCAGAACCGCCAC

B43 CACGCATAAGAAAGGAACAACTAAGTCTTTCC

B44 ATTGTGTCTCAGCAGCGAAAGACACCATCGCC

B45 TTAATAAAACGAACTAACCGAACTGACCAACTCCTGATAA

B46 AGGTTTAGTACCGCCATGAGTTTCGTCACCAGGATCTAAA

B47 GTTTTGTCAGGAATTGCGAATAATCCGACAAT

B48 GACAACAAGCATCGGAACGAGGGTGAGATTTG

B49 TATCATCGTTGAAAGAGGACAGATGGAAGAAAAATCTACG

B50 AGCGTAACTACAAACTACAACGCCTATCACCGTACTCAGG

B51 TAGTTGCGAATTTTTTCACGTTGATCATAGTT

B52 GTACAACGAGCAACGGCTACAGAGGATACCGA

B53 ACCAGTCAGGACGTTGGAACGGTGTACAGACCGAAACAAA

B54 ACAGACAGCCCAAATCTCCAAAAAAAAATTTCTTA

B55 AACAGCTTGCTTTGAGGACTAAAGCGATTATA

B56 CCAAGCGCAGGCGCATAGGCTGGCAGAACTGGCTCATTAT

B57 CGAGGTGAGGCTCCAAAAGGAGCC

B58 ACCCCCAGACTTTTTCATGAGGAACTTGCTTT

B59 ACCTTATGCGATTTTATGACCTTCATCAAGAGCATCTTTG

B60 CGGTTTATCAGGTTTCCATTAAACGGGAATACACT

B61 AAAACACTTAATCTTGACAAGAACTTAATCATTGTGAATT

B62 GGCAAAAGTAAAATACGTAATGCC

B63-CD44 aptamer ACCGGGCGTACACCGTCGCGGCACATGTCTGAATGCGTTTAGTCTCTGTGAAAAAATGGTTTAATTTCAACTCGGATATTCATTACCCACGAAAGA

B64 ACCAACCTAAAAAATCAACGTAACAAATAAATTGGGCTTGAGAS32

B65 CCTGACGAGAAACACCAGAACGAGTAGGCTGCTCATTCAGTGA

Link-A1C TTAATTAATTTTTTACCATATCAAA

Link-A2C TTAATTTCATCTTAGACTTTACAA

Link-A3C CTGTCCAGACGTATACCGAACGA

Link-A4C TCAAGATTAGTGTAGCAATACT

Link-B1A TGTAGCATTCCTTTTATAAACAGTT

Link-B2A TTTAATTGTATTTCCACCAGAGCC

Link-B3A ACTACGAAGGCTTAGCACCATTA

Link-B4A ATAAGGCTTGCAACAAAGTTAC

Link-C1B GTGGGAACAAATTTCTATTTTTGAG

Link-C2B CGGTGCGGGCCTTCCAAAAACATT

Link-C3B ATGAGTGAGCTTTTAAATATGCA

Link-C4B ACTATTAAAGAGGATAGCGTCC

Loop GCGCTTAATGCGCCGCTACAGGGC

C01 TCGGGAGATATACAGTAACAGTACAAATAATT

C02 CCTGATTAAAGGAGCGGAATTATCTCGGCCTC

C03 GCAAATCACCTCAATCAATATCTGCAGGTCGA

C04 CGACCAGTACATTGGCAGATTCACCTGATTGC

C05 TGGCAATTTTTAACGTCAGATGAAAACAATAACGGATTCG

C06 AAGGAATTACAAAGAAACCACCAGTCAGATGA

C07 GGACATTCACCTCAAATATCAAACACAGTTGA

C08 TTGACGAGCACGTATACTGAAATGGATTATTTAATAAAAG

C09 CCTGATTGCTTTGAATTGCGTAGATTTTCAGGCATCAATA

C10 TAATCCTGATTATCATTTTGCGGAGAGGAAGG

C11 TTATCTAAAGCATCACCTTGCTGATGGCCAAC

C12 AGAGATAGTTTGACGCTCAATCGTACGTGCTTTCCTCGTT

C13 GATTATACACAGAAATAAAGAAATACCAAGTTACAAAATC

C14 TAGGAGCATAAAAGTTTGAGTAACATTGTTTG

C15 TGACCTGACAAATGAAAAATCTAAAATATCTT

C16 AGAATCAGAGCGGGAGATGGAAATACCTACATAACCCTTC

C17 GCGCAGAGGCGAATTAATTATTTGCACGTAAATTCTGAAT

C18 AATGGAAGCGAACGTTATTAATTTCTAACAAC

C19 TAATAGATCGCTGAGAGCCAGCAGAAGCGTAA

C20 GAATACGTAACAGGAAAAACGCTCCTAAACAGGAGGCCGA

C21 TCAATAGATATTAAATCCTTTGCCGGTTAGAACCT

C22 CAATATTTGCCTGCAACAGTGCCATAGAGCCG

C23 TTAAAGGGATTTTAGATACCGCCAGCCATTGCGGCACAGA

C24 ACAATTCGACAACTCGTAATACAT

C25 TTGAGGATGGTCAGTATTAACACCTTGAATGG

C26 CTATTAGTATATCCAGAACAATATCAGGAACGGTACGCCA

C27 CGCGAACTAAAACAGAGGTGAGGCTTAGAAGTATT

C28 GAATCCTGAGAAGTGTATCGGCCTTGCTGGTACTTTAATG

C29 ACCACCAGCAGAAGATGATAGCCC

C30 TAAAACATTAGAAGAACTCAAACTTTTTATAATCAGTGAGS33

C31-CD44 aptamer ACCGGGCGTACACCGTCGCGGCACATGTCTGAATGCGTTTAGTCTCTGTGAAAAAGCCACCGAGTAAAAGAACATCACTTGCCTGAGCGCCATTAAAA

C32 TCTTTGATTAGTAATAGTCTGTCCATCACGCAAATTAACCGTT

C33 CGCGTCTGATAGGAACGCCATCAACTTTTACA

C34 AGGAAGATGGGGACGACGACAGTAATCATATT

C35 CTCTAGAGCAAGCTTGCATGCCTGGTCAGTTG

C36 CCTTCACCGTGAGACGGGCAACAGCAGTCACA

C37 CGAGAAAGGAAGGGAAGCGTACTATGGTTGCT

C38 GCTCATTTTTTAACCAGCCTTCCTGTAGCCAGGCATCTGC

C39 CAGTTTGACGCACTCCAGCCAGCTAAACGACG

C40 GCCAGTGCGATCCCCGGGTACCGAGTTTTTCT

C41 TTTCACCAGCCTGGCCCTGAGAGAAAGCCGGCGAACGTGG

C42 GTAACCGTCTTTCATCAACATTAAAATTTTTGTTAAATCA

C43 ACGTTGTATTCCGGCACCGCTTCTGGCGCATC

C44 CCAGGGTGGCTCGAATTCGTAATCCAGTCACG

C45 TAGAGCTTGACGGGGAGTTGCAGCAAGCGGTCATTGGGCG

C46 GTTAAAATTCGCATTAATGTGAGCGAGTAACACACGTTGG

C47 TGTAGATGGGTGCCGGAAACCAGGAACGCCAG

C48 GGTTTTCCATGGTCATAGCTGTTTGAGAGGCG

C49 GTTTGCGTCACGCTGGTTTGCCCCAAGGGAGCCCCCGATT

C50 GGATAGGTACCCGTCGGATTCTCCTAAACGTTAATATTTT

C51 AGTTGGGTCAAAGCGCCATTCGCCCCGTAATG

C52 CGCGCGGGCCTGTGTGAAATTGTTGGCGATTA

C53 CTAAATCGGAACCCTAAGCAGGCGAAAATCCTTCGGCCAA

C54 CGGCGGATTGAATTCAGGCTGCGCAACGGGGGATG

C55 TGCTGCAAATCCGCTCACAATTCCCAGCTGCA

C56 TTAATGAAGTTTGATGGTGGTTCCGAGGTGCCGTAAAGCA

C57 TGGCGAAATGTTGGGAAGGGCGAT

C58 TGTCGTGCACACAACATACGAGCCACGCCAGC

C59 CAAGTTTTTTGGGGTCGAAATCGGCAAAATCCGGGAAACC

C60 TCTTCGCTATTGGAAGCATAAAGTGTATGCCCGCT

C61 TTCCAGTCCTTATAAATCAAAAGAGAACCATCACCCAAAT

C62 GCGCTCACAAGCCTGGGGTGCCTA

C63-CD44 aptamer ACCGGGCGTACACCGTCGCGGCACATGTCTGAATGCGTTTAGTCTCTGTGAAAAACGATGGCCCACTACGTATAGCCCGAGATAGGGATTGCGTT

C64 AACTCACATTATTGAGTGTTGTTCCAGAAACCGTCTATCAGGG

C65 ACGTGGACTCCAACGTCAAAGGGCGAATTTGGAACAAGAGTCC

**Table S4.** DNA sequences of the non-targeted triangle.


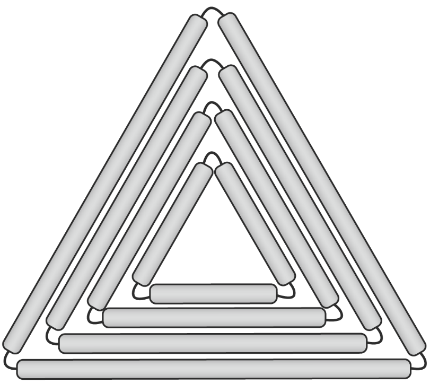


Name Sequences (5’ to 3’)

A01 CGGGGTTTCCTCAAGAGAAGGATTTTGAATTA

A02 AGCGTCATGTCTCTGAATTTACCGACTACCTT

A03 TTCATAATCCCCTTATTAGCGTTTTTCTTACC

A04 ATGGTTTATGTCACAATCAATAGATATTAAAC

A05 TTTGATGATTAAGAGGCTGAGACTTGCTCAGTACCAGGCG

A06 CCGGAACCCAGAATGGAAAGCGCAACATGGCT

A07 AAAGACAACATTTTCGGTCATAGCCAAAATCA

A08 GACGGGAGAATTAACTCGGAATAAGTTTATTTCCAGCGCC

A09 GATAAGTGCCGTCGAGCTGAAACATGAAAGTATACAGGAG

A10 TGTACTGGAAATCCTCATTAAAGCAGAGCCAC

A11 CACCGGAAAGCGCGTTTTCATCGGAAGGGCGA

A12 CATTCAACAAACGCAAAGACACCAGAACACCCTGAACAAA

A13 TTTAACGGTTCGGAACCTATTATTAGGGTTGATATAAGTA

A14 CTCAGAGCATATTCACAAACAAATTAATAAGT

A15 GGAGGGAATTTAGCGTCAGACTGTCCGCCTCC

A16 GTCAGAGGGTAATTGATGGCAACATATAAAAGCGATTGAG

A17 TAGCCCGGAATAGGTGAATGCCCCCTGCCTATGGTCAGTG

A18 CCTTGAGTCAGACGATTGGCCTTGCGCCACCC

A19 TCAGAACCCAGAATCAAGTTTGCCGGTAAATA

A20 TTGACGGAAATACATACATAAAGGGCGCTAATATCAGAGA

A21 CAGAGCCAGGAGGTTGAGGCAGGTAACAGTGCCCG

A22 ATTAAAGGCCGTAATCAGTAGCGAGCCACCCT

A23 GATAACCCACAAGAATGTTAGCAAACGTAGAAAATTATTC

A24 GCCGCCAGCATTGACACCACCCTC

A25 AGAGCCGCACCATCGATAGCAGCATGAATTAT

A26 CACCGTCACCTTATTACGCAGTATTGAGTTAAGCCCAATA

A27 AGCCATTTAAACGTCACCAATGAACACCAGAACCA

A28 ATAAGAGCAAGAAACATGGCATGATTAAGACTCCGACTTG

A29 CCATTAGCAAGGCCGGGGGAATTA

A30 GAGCCAGCGAATACCCAAAAGAACATGAAATAGCAATAGC

A31 TATCTTACCGAAGCCCAAACGCAATAATAACGAAAATCACCAG

A32 CAGAAGGAAACCGAGGTTTTTAAGAAAAGTAAGCAGATAGCCG

A33 CCTTTTTTCATTTAACAATTTCATAGGATTAG

A34 TTTAACCTATCATAGGTCTGAGAGTTCCAGTA

A35 AGTATAAAATATGCGTTATACAAAGCCATCTT

A36 CAAGTACCTCATTCCAAGAACGGGAAATTCAT

A37 AGAGAATAACATAAAAACAGGGAAGCGCATTA

A38 AAAACAAAATTAATTAAATGGAAACAGTACATTAGTGAAT

A39 TTATCAAACCGGCTTAGGTTGGGTAAGCCTGT

A40 TTAGTATCGCCAACGCTCAACAGTCGGCTGTC

A41 TTTCCTTAGCACTCATCGAGAACAATAGCAGCCTTTACAGS30

A42 AGAGTCAAAAATCAATATATGTGATGAAACAAACATCAAG

A43 ACTAGAAATATATAACTATATGTACGCTGAGA

A44 TCAATAATAGGGCTTAATTGAGAATCATAATT

A45 AACGTCAAAAATGAAAAGCAAGCCGTTTTTATGAAACCAA

A46 GAGCAAAAGAAGATGAGTGAATAACCTTGCTTATAGCTTA

A47 GATTAAGAAATGCTGATGCAAATCAGAATAAA

A48 CACCGGAATCGCCATATTTAACAAAATTTACG

A49 AGCATGTATTTCATCGTAGGAATCAAACGATTTTTTGTTT

A50 ACATAGCGCTGTAAATCGTCGCTATTCATTTCAATTACCT

A51 GTTAAATACAATCGCAAGACAAAGCCTTGAAA

A52 CCCATCCTCGCCAACATGTAATTTAATAAGGC

A53 TCCCAATCCAAATAAGATTACCGCGCCCAATAAATAATAT

A54 TCCCTTAGAATAACGCGAGAAAACTTTTACCGACC

A55 GTGTGATAAGGCAGAGGCATTTTCAGTCCTGA

A56 ACAAGAAAGCAAGCAAATCAGATAACAGCCATATTATTTA

A57 GTTTGAAATTCAAATATATTTTAG

A58 AATAGATAGAGCCAGTAATAAGAGATTTAATG

A59 GCCAGTTACAAAATAATAGAAGGCTTATCCGGTTATCAAC

A60 TTCTGACCTAAAATATAAAGTACCGACTGCAGAAC

A61 GCGCCTGTTATTCTAAGAACGCGATTCCAGAGCCTAATTT

A62 TCAGCTAAAAAAGGTAAAGTAATT

A63 ACGCTAACGAGCGTCTGGCGTTTTAGCGAACCCAACATGT

A64 ACGACAATAAATCCCGACTTGCGGGAGATCCTGAATCTTACCA

A65 TGCTATTTTGCACCCAGCTACAATTTTGTTTTGAAGCCTTAAA

B01 TCATATGTGTAATCGTAAAACTAGTCATTTTC

B02 GTGAGAAAATGTGTAGGTAAAGATACAACTTT

B03 GGCATCAAATTTGGGGCGCGAGCTAGTTAAAG

B04 TTCGAGCTAAGACTTCAAATATCGGGAACGAG

B05 ACAGTCAAAGAGAATCGATGAACGACCCCGGTTGATAATC

B06 ATAGTAGTATGCAATGCCTGAGTAGGCCGGAG

B07 AACCAGACGTTTAGCTATATTTTCTTCTACTA

B08 GAATACCACATTCAACTTAAGAGGAAGCCCGATCAAAGCG

B09 AGAAAAGCCCCAAAAAGAGTCTGGAGCAAACAATCACCAT

B10 CAATATGACCCTCATATATTTTAAAGCATTAA

B11 CATCCAATAAATGGTCAATAACCTCGGAAGCA

B12 AACTCCAAGATTGCATCAAAAAGATAATGCAGATACATAA

B13 CGTTCTAGTCAGGTCATTGCCTGACAGGAAGATTGTATAA

B14 CAGGCAAGATAAAAATTTTTAGAATATTCAAC

B15 GATTAGAGATTAGATACATTTCGCAAATCATA

B16 CGCCAAAAGGAATTACAGTCAGAAGCAAAGCGCAGGTCAG

B17 GCAAATATTTAAATTGAGATCTACAAAGGCTACTGATAAA

B18 TTAATGCCTTATTTCAACGCAAGGGCAAAGAA

B19 TTAGCAAATAGATTTAGTTTGACCAGTACCTT

B20 TAATTGCTTTACCCTGACTATTATGAGGCATAGTAAGAGCS31

B21 ATAAAGCCTTTGCGGGAGAAGCCTGGAGAGGGTAG

B22 TAAGAGGTCAATTCTGCGAACGAGATTAAGCA

B23 AACACTATCATAACCCATCAAAAATCAGGTCTCCTTTTGA

B24 ATGACCCTGTAATACTTCAGAGCA

B25 TAAAGCTATATAACAGTTGATTCCCATTTTTG

B26 CGGATGGCACGAGAATGACCATAATCGTTTACCAGACGAC

B27 TAATTGCTTGGAAGTTTCATTCCAAATCGGTTGTA

B28 GATAAAAACCAAAATATTAAACAGTTCAGAAATTAGAGCT

B29 ACTAAAGTACGGTGTCGAATATAA

B30 TGCTGTAGATCCCCCTCAAATGCTGCGAGAGGCTTTTGCA

B31 AAAGAAGTTTTGCCAGCATAAATATTCATTGACTCAACATGTT

B32 AATACTGCGGAATCGTAGGGGGTAATAGTAAAATGTTTAGACT

B33 AGGGATAGCTCAGAGCCACCACCCCATGTCAA

B34 CAACAGTTTATGGGATTTTGCTAATCAAAAGG

B35 GCCGCTTTGCTGAGGCTTGCAGGGGAAAAGGT

B36 GCGCAGACTCCATGTTACTTAGCCCGTTTTAA

B37 ACAGGTAGAAAGATTCATCAGTTGAGATTTAG

B38 CCTCAGAACCGCCACCCAAGCCCAATAGGAACGTAAATGA

B39 ATTTTCTGTCAGCGGAGTGAGAATACCGATAT

B40 ATTCGGTCTGCGGGATCGTCACCCGAAATCCG

B41 CGACCTGCGGTCAATCATAAGGGAACGGAACAACATTATT

B42 AGACGTTACCATGTACCGTAACACCCCTCAGAACCGCCAC

B43 CACGCATAAGAAAGGAACAACTAAGTCTTTCC

B44 ATTGTGTCTCAGCAGCGAAAGACACCATCGCC

B45 TTAATAAAACGAACTAACCGAACTGACCAACTCCTGATAA

B46 AGGTTTAGTACCGCCATGAGTTTCGTCACCAGGATCTAAA

B47 GTTTTGTCAGGAATTGCGAATAATCCGACAAT

B48 GACAACAAGCATCGGAACGAGGGTGAGATTTG

B49 TATCATCGTTGAAAGAGGACAGATGGAAGAAAAATCTACG

B50 AGCGTAACTACAAACTACAACGCCTATCACCGTACTCAGG

B51 TAGTTGCGAATTTTTTCACGTTGATCATAGTT

B52 GTACAACGAGCAACGGCTACAGAGGATACCGA

B53 ACCAGTCAGGACGTTGGAACGGTGTACAGACCGAAACAAA

B54 ACAGACAGCCCAAATCTCCAAAAAAAAATTTCTTA

B55 AACAGCTTGCTTTGAGGACTAAAGCGATTATA

B56 CCAAGCGCAGGCGCATAGGCTGGCAGAACTGGCTCATTAT

B57 CGAGGTGAGGCTCCAAAAGGAGCC

B58 ACCCCCAGACTTTTTCATGAGGAACTTGCTTT

B59 ACCTTATGCGATTTTATGACCTTCATCAAGAGCATCTTTG

B60 CGGTTTATCAGGTTTCCATTAAACGGGAATACACT

B61 AAAACACTTAATCTTGACAAGAACTTAATCATTGTGAATT

B62 GGCAAAAGTAAAATACGTAATGCC

B63 TGGTTTAATTTCAACTCGGATATTCATTACCCACGAAAGA

B64 ACCAACCTAAAAAATCAACGTAACAAATAAATTGGGCTTGAGAS32

B65 CCTGACGAGAAACACCAGAACGAGTAGGCTGCTCATTCAGTGA

Link-A1C TTAATTAATTTTTTACCATATCAAA

Link-A2C TTAATTTCATCTTAGACTTTACAA

Link-A3C CTGTCCAGACGTATACCGAACGA

Link-A4C TCAAGATTAGTGTAGCAATACT

Link-B1A TGTAGCATTCCTTTTATAAACAGTT

Link-B2A TTTAATTGTATTTCCACCAGAGCC

Link-B3A ACTACGAAGGCTTAGCACCATTA

Link-B4A ATAAGGCTTGCAACAAAGTTAC

Link-C1B GTGGGAACAAATTTCTATTTTTGAG

Link-C2B CGGTGCGGGCCTTCCAAAAACATT

Link-C3B ATGAGTGAGCTTTTAAATATGCA

Link-C4B ACTATTAAAGAGGATAGCGTCC

Loop GCGCTTAATGCGCCGCTACAGGGC

C01 TCGGGAGATATACAGTAACAGTACAAATAATT

C02 CCTGATTAAAGGAGCGGAATTATCTCGGCCTC

C03 GCAAATCACCTCAATCAATATCTGCAGGTCGA

C04 CGACCAGTACATTGGCAGATTCACCTGATTGC

C05 TGGCAATTTTTAACGTCAGATGAAAACAATAACGGATTCG

C06 AAGGAATTACAAAGAAACCACCAGTCAGATGA

C07 GGACATTCACCTCAAATATCAAACACAGTTGA

C08 TTGACGAGCACGTATACTGAAATGGATTATTTAATAAAAG

C09 CCTGATTGCTTTGAATTGCGTAGATTTTCAGGCATCAATA

C10 TAATCCTGATTATCATTTTGCGGAGAGGAAGG

C11 TTATCTAAAGCATCACCTTGCTGATGGCCAAC

C12 AGAGATAGTTTGACGCTCAATCGTACGTGCTTTCCTCGTT

C13 GATTATACACAGAAATAAAGAAATACCAAGTTACAAAATC

C14 TAGGAGCATAAAAGTTTGAGTAACATTGTTTG

C15 TGACCTGACAAATGAAAAATCTAAAATATCTT

C16 AGAATCAGAGCGGGAGATGGAAATACCTACATAACCCTTC

C17 GCGCAGAGGCGAATTAATTATTTGCACGTAAATTCTGAAT

C18 AATGGAAGCGAACGTTATTAATTTCTAACAAC

C19 TAATAGATCGCTGAGAGCCAGCAGAAGCGTAA

C20 GAATACGTAACAGGAAAAACGCTCCTAAACAGGAGGCCGA

C21 TCAATAGATATTAAATCCTTTGCCGGTTAGAACCT

C22 CAATATTTGCCTGCAACAGTGCCATAGAGCCG

C23 TTAAAGGGATTTTAGATACCGCCAGCCATTGCGGCACAGA

C24 ACAATTCGACAACTCGTAATACAT

C25 TTGAGGATGGTCAGTATTAACACCTTGAATGG

C26 CTATTAGTATATCCAGAACAATATCAGGAACGGTACGCCA

C27 CGCGAACTAAAACAGAGGTGAGGCTTAGAAGTATT

C28 GAATCCTGAGAAGTGTATCGGCCTTGCTGGTACTTTAATG

C29 ACCACCAGCAGAAGATGATAGCCC

C30 TAAAACATTAGAAGAACTCAAACTTTTTATAATCAGTGAGS33

C31 GCCACCGAGTAAAAGAACATCACTTGCCTGAGCGCCATTAAAA

C32 TCTTTGATTAGTAATAGTCTGTCCATCACGCAAATTAACCGTT

C33 CGCGTCTGATAGGAACGCCATCAACTTTTACA

C34 AGGAAGATGGGGACGACGACAGTAATCATATT

C35 CTCTAGAGCAAGCTTGCATGCCTGGTCAGTTG

C36 CCTTCACCGTGAGACGGGCAACAGCAGTCACA

C37 CGAGAAAGGAAGGGAAGCGTACTATGGTTGCT

C38 GCTCATTTTTTAACCAGCCTTCCTGTAGCCAGGCATCTGC

C39 CAGTTTGACGCACTCCAGCCAGCTAAACGACG

C40 GCCAGTGCGATCCCCGGGTACCGAGTTTTTCT

C41 TTTCACCAGCCTGGCCCTGAGAGAAAGCCGGCGAACGTGG

C42 GTAACCGTCTTTCATCAACATTAAAATTTTTGTTAAATCA

C43 ACGTTGTATTCCGGCACCGCTTCTGGCGCATC

C44 CCAGGGTGGCTCGAATTCGTAATCCAGTCACG

C45 TAGAGCTTGACGGGGAGTTGCAGCAAGCGGTCATTGGGCG

C46 GTTAAAATTCGCATTAATGTGAGCGAGTAACACACGTTGG

C47 TGTAGATGGGTGCCGGAAACCAGGAACGCCAG

C48 GGTTTTCCATGGTCATAGCTGTTTGAGAGGCG

C49 GTTTGCGTCACGCTGGTTTGCCCCAAGGGAGCCCCCGATT

C50 GGATAGGTACCCGTCGGATTCTCCTAAACGTTAATATTTT

C51 AGTTGGGTCAAAGCGCCATTCGCCCCGTAATG

C52 CGCGCGGGCCTGTGTGAAATTGTTGGCGATTA

C53 CTAAATCGGAACCCTAAGCAGGCGAAAATCCTTCGGCCAA

C54 CGGCGGATTGAATTCAGGCTGCGCAACGGGGGATG

C55 TGCTGCAAATCCGCTCACAATTCCCAGCTGCA

C56 TTAATGAAGTTTGATGGTGGTTCCGAGGTGCCGTAAAGCA

C57 TGGCGAAATGTTGGGAAGGGCGAT

C58 TGTCGTGCACACAACATACGAGCCACGCCAGC

C59 CAAGTTTTTTGGGGTCGAAATCGGCAAAATCCGGGAAACC

C60 TCTTCGCTATTGGAAGCATAAAGTGTATGCCCGCT

C61 TTCCAGTCCTTATAAATCAAAAGAGAACCATCACCCAAAT

C62 GCGCTCACAAGCCTGGGGTGCCTA

C63 CGATGGCCCACTACGTATAGCCCGAGATAGGGATTGCGTT

C64 AACTCACATTATTGAGTGTTGTTCCAGAAACCGTCTATCAGGG

C65 ACGTGGACTCCAACGTCAAAGGGCGAATTTGGAACAAGAGTCC
